# Immune cell status, cardiorespiratory fitness and body composition among breast cancer survivors and healthy women: a cross sectional study

**DOI:** 10.3389/fphys.2023.1107070

**Published:** 2023-06-01

**Authors:** Ainhoa Arana Echarri, Lauren Struszczak, Mark Beresford, John P. Campbell, Robert H. Jones, Dylan Thompson, James E. Turner

**Affiliations:** ^1^ Department for Health, University of Bath, Bath, United Kingdom; ^2^ Department for Oncology and Haematology, Royal United Hospitals Bath NHS Trust, Bath, United Kingdom; ^3^ Velindre Cancer Centre and Cardiff University, Cardiff, United Kingdom; ^4^ School of Sport, Exercise and Rehabilitation Sciences, College of Life and Environmental Sciences, University of Birmingham, Birmingham, United Kingdom

**Keywords:** breast cancer treatment, immune profiles, leukocytes, lymphocytes, lifestyle, exercise, cardiorespiratory fitness, body composition

## Abstract

**Methods:** We examined whether immune cell profiles differ between healthy women (*n* = 38) and breast cancer survivors (*n* = 27) within 2 years of treatment, and whether any group-differences were influenced by age, cytomegalovirus infection, cardiorespiratory fitness and body composition. Using flow cytometry, CD4+ and CD8+ T cell subsets, including naïve (NA), central memory (CM) and effector cells (EM and EMRA) were identified using CD27/CD45RA. Activation was measured by HLA-DR expression. Stem cell-like memory T cells (TSCMs) were identified using CD95/CD127. B cells, including plasmablasts, memory, immature and naïve cells were identified using CD19/CD27/CD38/CD10. Effector and regulatory Natural Killer cells were identified using CD56/CD16.

**Results:** Compared to healthy women, CD4+ CM were +Δ21% higher among survivors (*p* = 0.028) and CD8+ NA were −Δ25% lower (*p* = 0.034). Across CD4+ and CD8+ subsets, the proportion of activated (HLA-DR+) cells was +Δ31% higher among survivors: CD4+ CM (+Δ25%), CD4+ EM (+Δ32%) and CD4+ EMRA (+Δ43%), total CD8^+^ (+Δ30%), CD8+ EM (+Δ30%) and CD8+ EMRA (+Δ25%) (*p* < 0.046). The counts of immature B cells, NK cells and CD16+ NK effector cells were higher among survivors (+Δ100%, +Δ108% and +Δ143% respectively, *p* < 0.04). Subsequent analyses examined whether statistically significant differences in participant characteristics, influenced immunological differences between groups. Compared to healthy women, survivors were older (56 ± 6 y vs. 45 ± 11 y), had lower cardiorespiratory fitness ( $\dot{V}O_{2max}$ mL kg^−1^ min^−1^: 28.8 ± 5.0 vs. 36.2 ± 8.5), lower lean mass (42.3 ± 5.0 kg vs. 48.4 ± 15.8 kg), higher body fat (36.3% ± 5.3% vs. 32.7% ± 6.4%) and higher fat mass index (FMI kg/m^2^: 9.5 ± 2.2 vs. 8.1 ± 2.7) (all *p* < 0.033). Analysis of covariance revealed divergent moderating effects of age, CMV serostatus, cardiorespiratory fitness and body composition on the differences in immune cell profiles between groups, depending on the cell type examined. Moreover, across all participants, fat mass index was positively associated with the proportion of HLA-DR+ CD4+ EMRA and CD8+ EM/EMRA T cells (Pearson correlation: *r* > 0.305, *p* < 0.019). The association between fat mass index and HLA-DR+ CD8+ EMRA T cells withstood statistical adjustment for all variables, including age, CMV serostatus, lean mass and cardiorespiratory fitness, potentially implicating these cells as contributors to inflammatory/immune-dysfunction in overweight/obesity.

## 1 Introduction

The numbers and characteristics of leukocyte subtypes assessed in blood prior to cancer therapy have been linked to treatment responses, disease progression, mortality and recurrence (Bassani et al., 2019; Palazón-Carrión et al., 2021; Tay et al., 2021; Xie et al., 2021). For example, it has been shown among gastric cancer patients prior to radical gastrectomy, that lower lymphocyte counts and higher monocyte counts were associated with poor prognosis and lower cumulative survival (Feng et al., 2018). Similarly, among patients with recurrent ovarian cancer, low NK cell counts prior to palliative chemotherapy were associated with an unfavourable prognosis and lower overall survival compared to patients with higher counts (Henriksen et al., 2020). Further, among patients with non-small cell lung cancer, those with a higher ratio of central memory to effector memory CD4+ and CD8+ T cells prior to receiving anti-PD1 therapy had longer progression free survival compared to patients with lower ratios (Manjarrez-Orduño et al., 2018).

The numbers and characteristics of leukocyte subtypes in blood also change during cancer therapy and with disease progression. The mechanisms are thought to include direct effects of chemotherapeutic drugs and alterations to haematopoietic stem cells, the bone marrow niche and the thymus, in combination with inflammation and chronic stimulation by tumour antigens (Shao et al., 2013; Kinsella and Dudakov, 2020). Overall, cancer and anti-cancer treatments can exacerbate immunosenescence (Onyema et al., 2015; Bruni et al., 2019), potentially contributing to poor treatment outcomes and recurrence, by for example, impairing anti-tumour immunity and providing a “protective niche” for dormant tumour cells (Gilbert and Hemann, 2010; Sizova et al., 2018). Indeed, it has been shown among patients with brain cancer, sarcoma or Non-Hodgkin lymphoma, that high-dose sequential chemotherapy progressively reduced the number of CD4+ T cells, CD8+ T cells and B cells in blood, but increased the percentage of activated HLA-DR+ T cells (Mackall et al., 1994). Similarly, by the final cycle of dose-intense chemotherapy among patients with breast cancer, several cell types declined compared to pre-chemotherapy levels, including; B cells, CD4+ T cells, CD8+ T cells and NK cells, and the percentage of activated HLA-DR+ T cells increased (Hakim et al., 1997). In both studies, some of these therapy-induced changes in the immune profiles took months to return to baseline levels.

Although many studies report a decline in the counts of the major leukocyte sub-types following chemotherapy, and similar observations have been made with other forms of treatment, including radiotherapy (Mozaffari et al., 2007), some studies show different results. For example, early diagnosed older breast cancer patients showed increased frequencies of CD8+ T cells, their cytotoxic sub-types and CD4+ effector memory T cells after 3 months of chemotherapy, although the frequencies of CD8+ central memory T cells and the CD4+/CD8+ ratio decreased (Bailur et al., 2017). An explanation for disparate findings among studies showing *both* increases *and* decreases in cell counts and frequencies following cancer treatment is partly because not all studies employ detailed immunophenotyping of major leukocyte sub-types. In doing so, cells with specific functions can be quantified, providing a more detailed understanding and nuanced interpretation of the direction of changes to cell counts. However, a contributing explanation for different results between studies is that age and lifestyle factors–which are known to affect the numbers and characteristics of leukocyte sub-types in blood–are often not considered (Arana Echarri et al., 2021). These factors include age, regular exercise, cardiorespiratory fitness, and body composition.

Among healthy adults, major leukocyte subtypes have been compared between regular exercisers and non-exercisers across different age groups (Yan et al., 2001). Ageing was associated with a trend for a decline in percentage of T cells irrespective of exercise status, an accumulation of NK cells, which was greater among exercisers, and a higher percentage of B cells among all non-exercising groups irrespective of age (Yan et al., 2001). In a similar study, older regular cyclists were compared to age-matched older adults and younger adults who did not exercise regularly (Duggal et al., 2018). Ageing was associated with a decline in total T cells and an accumulation of CD8^+^ central memory and effector memory cells. However, the older cyclists exhibited a smaller accumulation of effector/memory cells than older non-exercisers. An age-associated decline in total B cells and naïve B cells and an accumulation of memory B cells was also shown, which was less pronounced among older cyclists (Duggal et al., 2018). The effects of ageing and regular exercise on leukocytes are less well established among patients with cancer. However, one study separated treatment-naïve older patients with Chronic Lymphocytic Leukaemia into fit and unfit groups following functional fitness tests, and found significantly lower frequencies of neutrophils and low-differentiated NK cells, significantly higher frequencies of mature NK cells, and a trend for lower CD8+ T cells and lower NK cells among fit patients (Sitlinger et al., 2021).

A physically active lifestyle can influence body composition. In turn, the proportion of body mass comprised of lean or fat tissue can independently influence the number and characteristics of leukocyte subtypes in blood. Given the rise in obesity-associated cancers, a focus of research has been on determining the effects that adiposity might have on cancer immune-surveillance (Conroy et al., 2016). In a study that controlled for a variety of factors (e.g., age, race, smoking and socioeconomic status), healthy women with a higher body mass index had a greater count of total leukocytes, lymphocytes, CD4+ T cells and CD8+ T cells (Womack et al., 2007). Other studies among overweight and obese people report changes in the T cell pool that are linked to immunosenescence, including an accumulation of effector memory and senescent T cells (Alam et al., 2012; Costanzo et al., 2015), even among children (Spielmann et al., 2014). In clinical settings among obese patients with colorectal cancer, it has been shown that T cell subsets involved in cancer immune-surveillance decline in blood (Donninelli et al., 2017). Mechanistically, most focus in cancer contexts has been on NK cells (Elaraby et al., 2021), given that many studies, among cancer free but overweight and obese people, have reported alterations in NK cell numbers, phenotype and function (Viel et al., 2017; Bähr et al., 2020; Campiotti et al., 2021). Indeed, in cancer settings, obesity induces lipid accumulation in NK cells, impairing their metabolism, trafficking and cytotoxicity against tumour targets (Michelet et al., 2018). Furthermore, although fat mass is an important contributor to immunosenescence (Trim et al., 2018), lean tissue can also influence the number and characteristics of leukocyte subtypes in blood. For example, a study of newly diagnosed Small Cell Lung Cancer patients showed that those with a depletion of muscle mass (also known as sarcopenia) had significantly lower lymphocyte counts and a higher neutrophil/lymphocyte ratio compared to non-sarcopenic patients (Kim et al., 2016). Similarly, a study of gastric cancer patients awaiting radical gastrectomy showed that patients with sarcopenia had significantly lower lymphocyte counts and significantly higher neutrophil/lymphocyte ratio (Lin et al., 2018). Further, among patients with colorectal cancer scheduled for surgery, pre-operation muscle depletion was associated with lower co-stimulatory capacity of their dendritic cells (Malietzis et al., 2016).

In summary, immune cell profiles in blood can influence clinical outcomes and can change with cancer treatment. If these profiles are assessed to stratify patients and inform treatment decisions or post-treatment care, age, lifestyle and other participant characteristics such as cardiorespiratory fitness and body composition should also be considered due to the impact that these factors can have on immune cells in blood (Arana Echarri et al., 2021). Thus, the aim of this study was to examine whether there are differences in blood immune cell profiles between healthy women and breast cancer survivors, and whether immune cell profiles were influenced by age, cytomegalovirus infection, cardiorespiratory fitness and body composition.

## 2 Materials and methods

### 2.1 Participants and experimental design

Participants were recruited by local advertisement or via a participating oncology clinic (Table 1), as part of two independent studies comprising either healthy women or female breast cancer survivors. Inclusion criteria for healthy women were; aged between 25–69 years, not pregnant, and free from any form of chronic disease (e.g., cardiovascular disease, type I or type II diabetes, or other autoimmune and inflammatory diseases). Inclusion criteria for survivors were; women aged between 35–69 years, who were post-menopausal (or who had not had a menstrual period for at least 1 year), and with a past diagnosis of non-metastatic non-bilateral stage I-III invasive breast cancer. Eligible survivors received their last treatment at least 2 months before enrolment, but no longer than 5 years prior, however women on long-term endocrine therapy were eligible. Survivors were free from any significant cardiac comorbidity, severe hypertension (>200/120 mmHg) or cardiovascular disease and did not have an active infection at the time of enrolment. Survivors self-reported not to undertake regular structured physical activity for more than 30 min on two or more occasions per week. In the present study, the immune profile of 38 healthy women was compared to 27 female breast cancer survivors.

**TABLE 1:** Participants.

	Healthy	Survivors	Patients	Univariate ANOVA
Sample size	38	27	5	
Age (years)	45 ± 11	56 ± 6***	44 ± 9	F_(1,63)_ = 25.914 *p* < 0.001 ƞ^2^ = 0.291
Height (m)	1.67 ± 0.75	1.63 ± 0.06**	1.66 ± 0.05	F_(1,63)_ = 7.042 *p* = 0.010 ƞ^2^ = 0.101
Body mass (kg)	70.4 ± 17.2	68.5 ± 9.9	73.1 ± 9.6	F_(1,63)_ = 0.081 *p* = 0.777 ƞ^2^ = 0.001
BMI (kg/m^2^)	25.1 ± 5.0	25.9 ± 3.4	26.7 ± 4.1	F_(1,63)_ = 1.057 *p* = 0.308 ƞ^2^ = 0.017
$\dot{V}O_{2}$ max (mL kg^−1^ min^−1^)	36.2 ± 8.5	28.8 ± 5.0***	27.3 ± 2.2	F_(1,63)_ = 17.858 *p* < 0.001 ƞ^2^ = 0.221
Body fat (%)	32.7 ± 6.4	36.3 ± 5.3*	38.9 ± 5.9	F_(1,63)_ = 5.462 *p* = 0.023 ƞ^2^ = 0.080
Lean mass (kg)	48.4 ± 15.8	42.3 ± 5.0*	43.1 ± 5.1	F_(1,63)_ = 4.753 *p* = 0.033 ƞ^2^ = 0.070
Fat mass (kg)	22.6 ± 7.9	25.2 ± 5.9	28.4 ± 7.3	F_(1,63)_ = 3.203 *p* = 0.078 ƞ^2^ = 0.048
Fat mass index (kg/m^2^)	8.1 ± 2.7	9.5 ± 2.2*	10.4 ± 3.0	F_(1,63)_ = 6.801 *p* = 0.011 ƞ^2^ = 0.097
CMV+ individuals	17 (44.7%)	12 (44.4%)	4 (80%)	F_(1,63)_ = 0.001 *p* = 0.982 ƞ^2^<0.001
CMV IgG (IU/ml)^a^	14.79 ± 6.44	17.28 ± 5.37	13.81 ± 10.11	F_(1,27)_ = 1.296 *p* = 0.265 ƞ^2^ = 0.046

Data are mean ± standard deviation (SD).

^a^ CMV IgG for CMV seropositive individuals. Univariate ANOVA was performed using log10 transformed data between healthy women and breast cancer survivors only. Data from breast cancer patients is shown for a qualitative comparison.

Statistical significance: *p < 0.05 **p < 0.01 ***p < 0.001. Abbreviations: BMI, body mass index; CMV, Cytomegalovirus; IgG, Immunoglobulin G; $\dot{V}O_{2}$ max, cardiorespiratory fitness.

Given that possible differences between groups could be driven by; 1) the past formation and presence of a tumour; 2) previous disease progression and chronic stimulation by tumour antigens and tumour-derived inflammation; or 3) the effects anti-cancer treatments can have on the immune system, we also recruited recently diagnosed female patients with biopsy-proven non-metastatic non-bilateral stage I–III invasive breast cancer, who had not received any form of treatment, as part of a preliminary analysis (*n* = 5). For inclusion, these patients were aged 25–69 years, had adequate renal, liver and bone marrow function, no significant cardiac comorbidity, a World Health Organisation performance status of 0–1 and were due to receive neoadjuvant chemotherapy for approximately 6 months prior to surgery. Thus, the group of patients help to initially isolate possible effects of (1) the formation and presence of a tumour; in the absence of long-term exposure to cancer cells or treatment. All participants provided informed consent and the studies were approved by NHS research ethics committees, reference numbers: 15/SW/0004 and 18/WA/0314.

### 2.2 Characteristics of breast cancer survivors and patients

Diagnostic and treatment history is shown in Table 2 (and Supplementary Table S1). Among survivors (*n* = 27), 22.2% of the participants had been diagnosed with ductal carcinoma *in situ* (DCIS) and 7.4% had a multifocal carcinoma. 74.1% participants were positive for hormone receptors (ER/PR+) and 7.4% were positive for HER2 (human epidermal growth factor receptor 2). Adjuvant therapies included radiotherapy (81.5%), endocrine therapy (66.7%), chemotherapy (33.3%) and/or anti-HER2+ therapy (7.4%). Endocrine therapy included treatment with letrozole, anastrozole or tamoxifen, and chemotherapy included treatment with either FEC-T (fluorouracil, epirubicin and cyclophosphamide for three cycles followed by Docetaxel for three cycles), FEC-TH (fluorouracil, epirubicin and cyclophosphamide for three cycles followed by docetaxel for three cycles, plus trastuzumab to treat HER2+ tumours) or AC-paclitaxel (doxorubicin, cyclophosphamide and paclitaxel weekly for 9–12 weeks). At recruitment, the average of time since diagnosis was 14 ± 6 months and the average time since treatment was 12 ± 6 months.

**TABLE 2:** Diagnostic and treatment information for breast cancer survivors and patients.

Clinical summary	% of group, average or duration (mean ± SD)	
**Diagnosis**	**Survivors**	**Patients**
Ductal carcinoma *in situ* (DCIS)	22.2% (6/27)	0% (0/5)
Multifocal carcinoma	7.4% (2/27)	0% (0/5)
Hormone expression (ER/PR+)	74.1% (20/27)	40% (2/5)
HER2 expression (HER2+)	7.4% (2/27)	20% (1/5)
Tumour grade (G)	3.7% (G1), 48.1% (G2), 25,9% (G3)	20% (G2), 80% (G3)
TNM scoring		
Tumour (T)	40.7% (T1), 29.6% (T2), 7.4% (T3)	—
Nodes (N)	66.7% (N0), 22.2% (N1), 7.4% (N2), 3.7% (N3)	40% (N0), 60% (N1)
Metastasis (M)	100% (M0)	100% (M0)
Average time since diagnosis	14 ± 6 months	—
**Treatment**	**Survivors**	**Patients**
Surgery	100% (27/27)	100% (5/5)
Chemotherapy	33.3% (9/27)	100% (5/5)
Radiotherapy	81.5% (22/27)	0% (0/5)
Hormone therapy	66.7% (18/27)	0% (0/5)
Anti- HER2+ therapy	7.4% (2/27)	20% (1/5)
Average time since treatment	12 ± 6 months	—

Data is expressed as percentage of participants or as mean ± SD. Grade refers to the histologic grade (G1, G2, G3, etc.). TNM staging defines the characteristics of the tumour 0 to 4 (T), 0 to 3 (N) and 0 (M). Abbreviations: DCIS, Ductal carcinoma *in situ*. HER2, Human epidermal growth factor receptor 2.

Among the patients scheduled to undergo treatment (*n* = 5), the majority (80%) had grade three tumours, involved lymph nodes (60%), and were ER negative (60%) and HER2 negative (80%). The chemotherapy combination patients were due to receive was either FEC-T (fluorouracil, epirubicin and cyclophosphamide for three cycles followed by docetaxel for three cycles), FEC-TPH (with pertuzumab and trastuzumab added for cycles 4–6) or FEC-paclitaxel (Fluorouracil, Epirubicin and Cyclophosphamide for three cycles followed by paclitaxel for three cycles).

### 2.3 Physiological characteristics of participants

Participants attended a research laboratory in the morning, fasted overnight since 22:00 and having refrained from exercise, caffeine or alcohol in the previous 24 h. Blood pressure was measured with an automated sphygmomanometer (Diagnostec EW3106, Panasonic, Japan) using the dominant arm among healthy women, or the contralateral arm to the affected breast among survivors and patients. Height was assessed with a wall mounted stadiometer (Holtain Ltd., United Kingdom) and body mass was measured without shoes using electronic scales (BC-543 Monitor, Tanita, Tokyo, Japan). Waist and hip circumferences were measured (in centimetres) with a tape (SECA 201, Hamburg, Germany). Dual energy X-ray absorptiometry (DEXA; QDR, Discovery W, Hologic, Bedford, United Kingdom) was used for body composition analysis. Cardiorespiratory fitness ( $\dot{V}O_{2}$ max) was assessed using a treadmill-based exercise test, consisting of walking at 5.8 kph at 0% gradient increasing by 3% every 5 min for a total of 20 min. Expired air samples were collected in the final minute of each stage using Douglas bags. Oxygen and Carbon dioxide concentrations were assessed (Servomex 5200; Sussex, United Kingdom). The volume of air was measured with a dry gas meter (Harvard Apparatus; Cambridge, United Kingdom). $\dot{V}O_{2}$ max was estimated using age (Bruce, 1971).

### 2.4 Blood sample collection and storage

After a 10-min seated rest, approximately 40 mL of blood was collected into a syringe containing sodium heparin (4 IU/mL) for preparation of peripheral blood mononuclear cells (PBMCs). PBMCs were isolated using density gradient centrifugation. An additional 10 mL of blood was collected into a potassium–ethylenediaminetetraacetic acid (K_3_–EDTA) tube (BD Vacutainer, BD Biosciences, Oxford, United Kingdom) and an anticoagulant-free serum separation tube for preparation of plasma and serum respectively. The tube for serum was left to clot for 30 mins at room temperature. Tubes for serum and plasma were centrifuged for 10 min at 2,000 × g and 4°C, and the supernatant was collected and stored at −80°C until analysis. To isolate PBMCs, blood was diluted 1:1 with sterile RPMI (Sigma-Aldrich, Guillingham, United Kingdom), layered onto Ficoll-Paque™ plus (GE Healthcare; Buckinghamshire, United Kingdom) and centrifuged at 500 × g at 21°C for 25 min. PBMCs were washed in RPMI by centrifuging at 400 × g at 21°C for 10 min and washed again in RPMI at 300 × g at 21°C for 7 min. Platelets were removed by washing in RPMI at 200 × g at 21°C for 7 min, and then cells were counted with Trypan blue (1.5% acetic acid) using a haemocytometer. PBMCs were resuspended in freezing mix (70% RPMI, 20% FCS and 10% DMSO), and frozen at −1°C/min in a freezing container (Mr Frosty, Nalgene) in a −80°C freezer.

### 2.5 Sample preparation and flow cytometry

PBMCs were cryopreserved for 16 ± 8 months and thawed rapidly in a 37°C water bath and washed in media (RPMI 10% FCS, 1% penicillin-streptomycin) by centrifuging at 300 × g at 21°C for 7 min. If clumping was visible, 0.3 µL of benzonase nuclease (250 U/µL, HC, Novogen) was added to the pellet and incubated for 5 min before washing with media and centrifuging at 300 × g at 21°C for 7 min. Cells were counted and rested overnight in media, at a concentration of 2 million cells per mL in tubes that enable gas exchange, at 37°C and 5% CO_2_.

Two antibody panels were prepared per sample (a T cell panel, and a B cell and Natural Killer cell panel). A subsequent control panel prepared with cells from each participant included isotype controls for assessment of T cell activation. Cells were stained with a Fixable Viability Stain (FVS) conjugated to v450 (Beckton Dickinson; Oxford, United Kingdom) and 400,000 cells were added to tubes for each panel. The T cell panel included anti-CD3—PERCP clone SK7, anti-CD4—PE-Cy7 clone L200, anti-CD8—APC clone SK1, anti-CD45RA—FITC clone HI100, anti-CD127—APC-Cy7 clone A019D5, anti-HLA-DR—V500 clone G46-6, anti-CD27—PE clone M-T271 and anti-CD95—BV605 clone DX2. The B cell/NK cell panel included anti-CD3—PE-Cy7 clone SK7, anti-CD19—PERCP clone 4G7, anti-CD10—APC-Cy7 clone HI10a, anti-CD27—APC clone L128, anti-CD38—FITC clone HB7, anti-CD16—V500 clone 3G8 and anti-CD56—PE clone B159. The isotype control panel included HLA DR—V500 isotype control (IgG2a, κ, clone G155-178) and anti-CD3—PERCP clone SK7. Antibodies were purchased from BD Biosciences (Beckton Dickinson; Oxford, United Kingdom) with the exception of anti-CD127—APC-Cy7, anti-CD95—BV605 and anti-CD10—APC-Cy7 (Biolegend, CA, United States).

Samples were analysed within 2 h of preparation using a FACSAria III flow cytometer (Beckton Dickinson; Oxford, United Kingdom). Voltages were optimised and maintained for all samples and all participants. Compensation was performed each day using single-stained tubes and positive and negative compensation beads (Beckton Dickinson; Oxford, United Kingdom) and parameters calculated automatically (BD FACS DIVA™, Beckton Dickinson; Oxford, United Kingdom). Acquisition flow rate was maintained across samples. Approximately 70,000–100,000 events (T cell panel), 50,000–75,000 events (B cell panel) and 15,000 events (isotype control tube) were recorded from the lymphocyte gate.

### 2.6 Flow cytometry analysis

Data were analysed using Flowjo v10.7.1 (FlowJo. LLC, BD Biosciences, Beckton Dickinson; Oxford, United Kingdom). After doublets were excluded, an initial plot was created with SSC (side scatter) versus FSC (forward scatter) (Supplementary Figures S1, S2, panel B). Lymphocytes and viable cells (typically >90%) were identified. CD3+ T cells were identified as CD4+ and CD8+ and further defined as Naïve (NA: CD27+ CD45RA+), Central Memory (CM: CD27+ CD45RA−), Effector Memory (EM: CD27− CD45RA−) and Effector Memory expressing CD45RA (EMRA: CD27− CD45RA+) (Supplementary Figure S1). Activated T cells were defined as HLA-DR+ using an isotype control. Median Fluorescence Intensity (MFI) was used to examine HLA-DR expression density. Stem cell like memory T cells (TSCMs) were identified as CD95+ CD127+ from the CD4+ and CD8+ Naïve T cell pools (Gattinoni et al., 2011; Schmueck-Henneresse et al., 2015; Curran et al., 2019). B cells (CD3− CD19+) were further defined into: Plasmablasts/Plasma cells (CD3− CD19+ CD27+ CD38+), Memory B cells (CD3− CD19+ CD27+ CD38−), Naïve B cells (CD3− CD19+ CD27− CD10−) and Immature B cells (CD3− CD19+ CD27− CD10+) (Supplementary Figure S2). Natural Killer cells (CD3− CD19− CD56+) were divided into Effector (CD3− CD19− CD56+ CD16+) and Regulatory cells (CD3− CD19− CD56+ CD16−) (Cooper et al., 2001; Björkström et al., 2016). Absolute cell counts were computed using a dual platform approach using the leukocyte differential determined in fresh whole K_3_–EDTA blood on the day of sampling (Sysmex Cell Counter Kx 21; Sysmex Europe, Germany). The CD4+/CD8+ ratio was calculated to examine inverted or high ratios (<1 or >2.5), which have been linked to immunosenescence and chronic inflammation (Ferguson et al., 1995; Strindhall et al., 2013; Tylutka et al., 2021). Normal ratios were assumed to be ≥1 and ≤2.5 (McBride and Striker, 2017).

### 2.7 Assessment of cytomegalovirus (CMV) serostatus

CMV-specific IgG was assessed in serum using enzyme-linked immunosorbent assays (ELISAs) according to manufacturer instructions (ENZY-WELL, Diesse Diagnostica, Italy). Absorbance at 450 nm was determined using a SPECTROstar Nano plate reader (BMG Labtech Ltd., United Kingdom). Values were blank corrected, and concentrations were calculated using a 4-parameter curve. CMV+ was considered to be >1.2 IU/mL, and CMV− was <0.8 IU/mL. Inter and intra assay variation was 1.74% and <5.2% respectively.

### 2.8 Statistical analysis

Statistical analyses were conducted between healthy women (*n* = 38) and survivors (*n* = 27). Given the small sample size of patients (*n* = 5), data were included for qualitative comparisons. Normal distribution was assessed using descriptive statistics and Shapiro Wilk and Kolmogorov-Smirnov tests. Most data were not normally distributed and therefore all data were log10 transformed and compared between groups using univariate analyses of variance (ANOVA). Levene’s tests were interpreted for homogeneity of variance between groups (*p* > 0.05). When participant characteristics (e.g., age and body composition) were significantly different between groups, these variables were examined as covariates using analyses of covariance (ANCOVA). Effect sizes were reported as eta squared (ƞ^2^), where, ƞ^2^ = 0.01 is a small effect, ƞ^2^ = 0.06 is a medium effect and ƞ^2^ = 0.14 is a large effect size (Cohen, 1988). When participant characteristics were statistically significant covariates or if the statistically significant differences in cell measurements between groups was lost in ANCOVA analyses, Pearson correlations were used to determine the direction of effect and statistically significant correlations were examined further using linear regression. Statistical significance was accepted at *p* < 0.05 and performed using SPSS v27 (IBM Corp.; New York, United States). Figures were created with GraphPad Prism v9.0.0 for Windows (GraphPad Software; CA, United States).

## 3 Results

### 3.1 Leukocyte counts were not different between breast cancer survivors and healthy women and both groups exhibited a similar T cell profile and T cell subset activation patterns

There were no significant differences in total leukocytes, lymphocytes, monocytes or neutrophils between healthy women and breast cancer survivors [Summary of ANOVAs: F_(1,63)_ < 0.630, *p* > 0.430, ƞ^2^<0.01] (Table 3). Breast cancer patients exhibited higher counts of all leukocytes compared to both healthy women and survivors.

**TABLE 3:** Leukocyte differential.

Cell count (x10^9^/L)	Healthy	Survivors	Patients	Univariate ANOVA
Total leukocytes	5.27 ± 1.52	5.06 ± 1.33	7.03 ± 2.02	F_(1,63)_ = 0.253 *p* = 0.617 ƞ^2^ = 0.004
Lymphocytes	1.55 ± 0.48	1.46 ± 0.38	1.72 ± 0.33	F_(1,63)_ = 0.630 *p* = 0.430 ƞ^2^ = 0.010
Monocytes	0.41 ± 0.20	0.41 ± 0.18	0.54 ± 0.21	F_(1,63)_ = 0.008 *p* = 0.927 ƞ^2^ = 0.000
Neutrophils	3.31 ± 1.19	3.17 ± 1.13	4.76 ± 1.86	F_(1,63)_ = 0.202 *p* = 0.655 ƞ^2^ = 0.003

Data are mean standard ± deviation (SD) for healthy women (*n* = 38), breast cancer survivors (*n* = 27) and breast cancer patients (*n* = 5). Univariate ANOVA was performed using log10 transformed data between healthy women and breast cancer survivors only. Data from breast cancer patients is shown for a qualitative comparison. Monocytes refer to the “Mixed cells” fraction from an automated haematology analyser: <10% correspond to basophils and eosinophils. Statistical significance was considered as *p* < 0.05.

**FIGURE 1:**
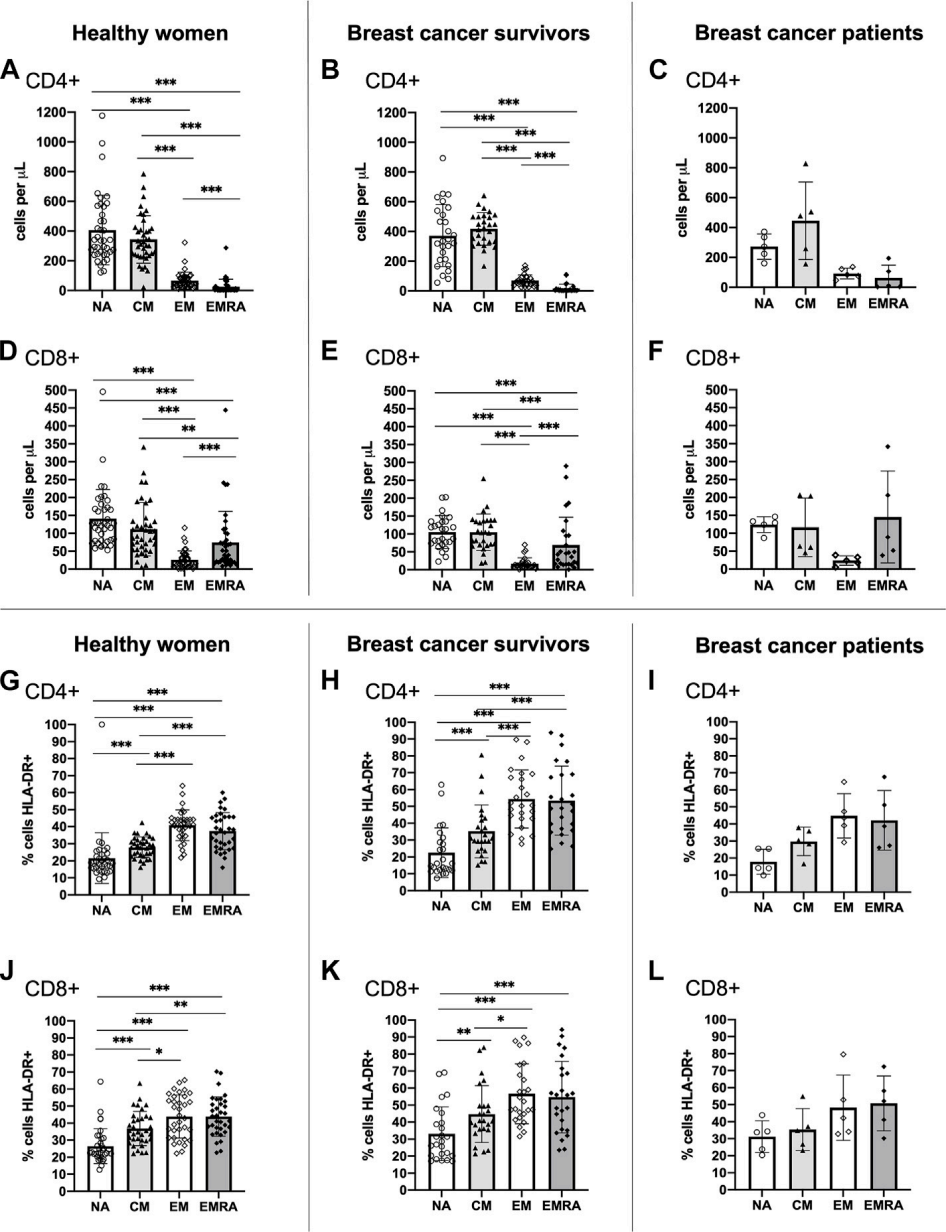
Absolute counts and proportion of activated cells among CD4+ and CD8+ T cell subsets within groups. Absolute counts **(A–F)** and proportion of activated cells expressing HLA-DR **(G–L)** for CD4+ and CD8+ T cell subsets: Naïve (NA), Central Memory (CM), Effector Memory (EM) and Effector Memory expressing CD45RA (EMRA) compared within study groups. Data are for healthy women (*n* = 38 for absolute counts, *n* = 34 for activation data), breast cancer survivors (*n* = 27 for absolute counts; *n* = 25 for activation data) and breast cancer patients (*n* = 5 for absolute counts and activation data). Data are mean ± standard deviation (SD). Statistical significance from Univariate ANOVAs using log10 transformed data between healthy and survivors is shown. *p < 0.05, **p < 0.01, ***p < 0.001.

A typical T cell profile and T cell subset activation pattern was exhibited by both breast cancer survivors and healthy women (Figure 1). Overall, both groups presented statistically significant differences in the number of cells within each T cell subset among both CD4+ [ANOVA: Healthy: F _(3,148)_ = 140.743, *p* < 0.001, ƞ^2^ = 0.740, Survivors: F_(3,104)_ = 159.808, *p* < 0.001, ƞ^2^ = 0.822] and CD8+ cells [ANOVA: Healthy: F_(3,148)_ = 37.070, *p* < 0.001, ƞ^2^ = 0.429, Survivors: F_(3,104)_ = 34.404, *p* < 0.001, ƞ^2^ = 0.498] (Figures 1A, B, D, E). For both groups, there were a greater number of CD4+ and CD8+ NA and CM cells compared to both effector memory subsets, EM and EMRA (all *p* < 0.01). Breast cancer patients exhibited a similar pattern to breast cancer survivors and healthy women (Figures 1C, F).

Both healthy women and breast cancer survivors exhibited statistically significant differences in the proportion of activated HLA-DR+ cells across the CD4+ T cell subsets [ANOVA: Healthy: F_(3,132)_ = 38.674, *p* < 0.001, ƞ^2^ = 0.468, Survivors: F_(3,96)_ = 28.862, *p* < 0.001, ƞ^2^ = 0.474] and the CD8+ T cell subsets [ANOVA: Healthy: F_(3,132)_ = 23.513, *p* < 0.001, ƞ^2^ = 0.348, Survivors: F_(3,96)_ = 11.842, *p* < 0.001, ƞ^2^ = 0.270] (Figures 1G, H, J, K). For both groups, the proportion of activated CD4+ and CD8+ EM and EMRA cells, was higher than the NA and CM subsets (all *p* < 0.05). A similar pattern was shown among breast cancer patients (Figures 1I, L). Analysis of HLA-DR expression density (i.e., median fluorescence intensity; MFI) showed almost identical results (**data not shown**).

### 3.2 CD4+ central memory T cells were higher and CD8+ naïve T cells were lower among breast cancer survivors compared to healthy women

**FIGURE 2:**
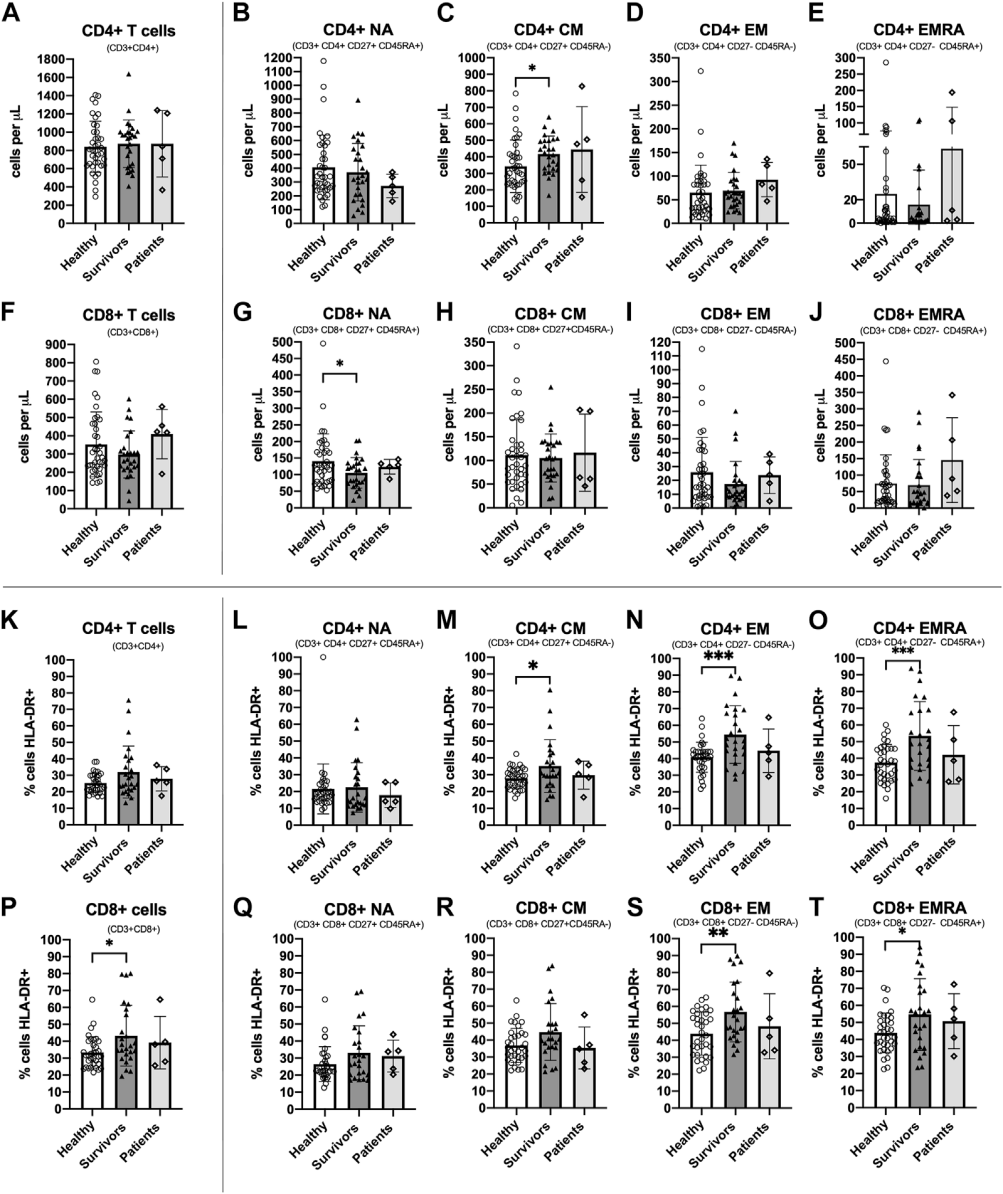
Absolute counts and proportion of activated cells among CD4+ and CD8+ T cell subsets among groups. Absolute counts **(A–J)** and proportion of activated cells expressing HLA-DR **(K–T)** for CD4+ and CD8+ T cell subsets: Naïve (NA), Central Memory (CM), Effector Memory (EM) and Effector Memory expressing CD45RA (EMRA) compared between study groups. Data are for healthy women (*n* = 38 for absolute counts; *n* = 34 for activation data), breast cancer survivors (*n* = 27 for absolute counts; *n* = 25 for activation data) and patients (*n* = 5 for absolute counts and activation data). Data are mean ± standard deviation (SD). Statistical significance from Univariate ANOVAs using log10 transformed data between healthy and survivors is shown. *p < 0.05, **p < 0.01, ***p < 0.001.

CD4+ CM T cells were higher [ANOVA F_(1,63)_ = 5.039, *p* = 0.028, ƞ^2^ = 0.074] and CD8+ NA T cells were lower [ANOVA F_(1,63)_ = 4.690, *p* = 0.034, ƞ^2^ = 0.069] among survivors compared to healthy women (Figures 2C, G). There were no statistically significant differences between groups among total CD4+ T cells or other subsets [Summary of ANOVAs: F_(1, 63)_<1.6, *p* > 0.211, ƞ^2^ < 0.025] (Figures 2A, B, D, E) or among total CD8+ T cells or other subsets [Summary of ANOVAs: F_(1, 63)_ < 2.018, *p* > 0.160, ƞ^2^ < 0.031] (Figures 2F, H, I, J). Breast cancer patients exhibited similar counts of CD4+ CM cells to survivors (although there was large variation between individuals) and similar counts of CD8+ NA T cells to both healthy women and survivors.

### 3.3 A greater proportion of CD4+ and CD8+ memory T cell subsets were activated among breast cancer survivors compared to healthy women

The proportion of cells expressing the activation marker HLA-DR was significantly higher among survivors compared to healthy women for CD4+ CM, CD4+ EM and CD4+ EMRA [Summary of ANOVAs: F_(1,57)_ > 4.160, *p* < 0.046, ƞ^2^ > 0.068] (Figures 2M–O) and for CD8+ T cells, CD8+ EM, and CD8+ EMRA [Summary of ANOVAs: F_(1, 57)_ > 4.131, *p* < 0.047, ƞ^2^ > 0.068] (Figures 2P, S, T). There were no statistically significant differences in the proportion of activated cells between groups among other CD4+ and CD8+ subsets [Summary of ANOVAs: F_(1,57)_ < 3.992, *p* > 0.051, ƞ^2^ < 0.065] (Figures 2K, L, Q, R). Among breast cancer patients, the proportion of activated CD4+ and CD8+ T cells and subsets was slightly lower than survivors and closer to the values for healthy women, but there was large inter-individual variation. Analysis of HLA-DR expression density showed almost identical results (**data not shown**).

### 3.4 The CD4+/CD8+ ratio tended to be higher among breast cancer survivors compared to healthy women

The CD4+/CD8+ ratio tended to be higher among survivors (3.7 ± 2.7) compared to healthy women (2.8 ± 1.5) but this difference was not statistically significant [ANOVA: F_(1, 63)_ = 2.945, *p* = 0.091, ƞ^2^ = 0.045] (Table 4). Overall, 52.6% of healthy women exhibited a normal ratio (≥1 and ≤2.5) compared to 33.3% of breast cancer survivors. In addition, 2.6% of healthy women exhibited an inverted ratio (<1) compared to 0% of breast cancer survivors. Finally, 44.7% of healthy women exhibited a high ratio (>2.5) compared to 66.7% of breast cancer survivors. Therefore, the percentage of breast cancer survivors with an inverted or higher ratio was higher than healthy women (Survivors: 66.7%, Healthy women: 47.3%; Pearson Chi square: 2.379 *p* = 0.138). Among breast cancer patients, 40% exhibited a normal CD4+/CD8+ ratio, 20% had an inverted ratio, and 40% had a high ratio.

**TABLE 4:** CD4+/CD8+ ratio and absolute counts for TSCMs, B cell subsets and NK cell subsets.

Subsets	Healthy	Survivors	Patients	Univariate ANOVA
CD4+/CD8+ ratio	2.82 ± 1.47	3.73 ± 2.66	2.68 ± 2.16	F_(1,63)_ = 2.945 *p* = 0.091 ƞ^2^ = 0.045
CD4+ TSCMs	4.00 ± 6.36	2.44 ± 1.12	1.52 ± 0.70	F_(1,63)_ = 0.473 *p* = 0.494 ƞ^2^ = 0.007
CD8+ TSCMs	0.50 ± 0.65	0.22 ± 0.42	0.49 ± 0.18	F_(1,63)_ = 3.531 *p* = 0.065 ƞ^2^ = 0.053
CD19+ total (B cells)	27.29 ± 23.89	39.96 ± 57.74	48.36 ± 53.24	F_(1,59)_ = 0.133 *p* = 0.717 ƞ^2^ = 0.002
Plasmablasts	1.17 ± 1.15	1.27 ± 1.28	3.35 ± 3.26	F_(1,59)_ = 0.021 *p* = 0.885 ƞ^2^ = 0.000
Memory B cells	3.91 ± 3.97	5.35 ± 8.60	15.31 ± 25.02	F_(1,59)_ = 0.006 *p* = 0.936 ƞ^2^ = 0.000
Immature B cells	0.71 ± 0.99	2.35 ± 4.79*	1.72 ± 2.52	F_(1,59)_ = 4.417 *p* = 0.040 ƞ^2^ = 0.070
Naive B cells	21.54 ± 19.67	30.81 ± 49.11	27.66 ± 22.71	F_(1,59)_ = 0.008 *p* = 0.929 ƞ^2^ = 0.000
CD56+ total (NK cells)	61.43 ± 55.28	127.31 ± 106.19**	93.31 ± 57.59	F_(1,59)_ = 11.403 *p* = 0.001 ƞ^2^ = 0.162
CD16+ Effector cells	43.66 ± 52.59	106.54 ± 98.05***	75.57 ± 56.31	F_(1,59)_ = 13.600 *p* < 0.001 ƞ^2^ = 0.187
CD16- Regulatory cells	17.71 ± 13.60	20.73 ± 13.35	17.74 ± 3.60	F_(1,59)_ = 1.840 *p* = 0.180 ƞ^2^ = 0.030

Data are mean standard ± deviation (SD) for healthy women (*n* = 38), breast cancer survivors (*n* = 27) and breast cancer patients (*n* = 5). B cells and NK cells are *n* = 35 for healthy women and n = 26 for breast cancer survivors. Univariate ANOVA was performed using log10 transformed data, between healthy women and breast cancer survivors. Data from breast cancer patients is shown for a qualitative comparison. Statistical significance: *p < 0.05 **p < 0.01 ***p < 0.001.

### 3.5 Stem cell-like memory T cells tended to be lower among breast cancer survivors compared to healthy women

There were no statistically significant differences in the number of CD4+ or CD8+ TSCMs between survivors and healthy women [Summary of ANOVAs: F_(1,63)_ < 3.531, *p* > 0.065, ƞ^2^ < 0.053] but cell numbers among survivors were approximately half of those exhibited by healthy women (CD4+ TSCMs: 4.00 ± 6.36 cells/ µ L vs. 2.44 ± 1.12 cells/ µ L CD8+ TSCMs: 0.50 ± 0.65 cells/ µ L vs. 0.22 ± 0.42 cells/ µ L) (Table 4). Breast cancer patients tended to exhibit lower CD4+ TSCM counts than both groups (CD4+ TSCMs: 1.52 ± 0.70 cells/ µ L) but CD8+ TSCMs tended to be higher among patients compared to survivors (CD8+ TSCMs: 0.49 ± 0.18 cells/ µ L) but similar to healthy women.

### 3.6 B cells and B cell subtypes tended to be higher among breast cancer survivors compared to healthy women

Survivors exhibited a greater number of immature B cells compared to healthy women (2.35 ± 4.79 cells/ µ L vs. 0.71 ± 0.99 cells/ µ L [ANOVA: F_(1,59)_ = 4.417, *p* = 0.040, ƞ^2^ = 0.070] and a tendency for higher counts in the other B cell subtypes (Table 4). There were no statistically significant differences in the number of total B cells and most other B cell subtypes between the groups [Summary of ANOVAs: F_(1,59)_ < 0.133, *p* > 0.717, ƞ^2^ < 0.002] (Table 4). Breast cancer patients tended to have total B cell and B cell subset counts that were closer to the counts exhibited by survivors and higher than healthy women.

### 3.7 NK cells were higher among breast cancer survivors compared to healthy women, driven by an expansion of effector cells

Survivors exhibited a greater number of total NK cells [127.31 ± 106.19 cells/μL vs. 61.43 ± 55.28 cells/μL. ANOVA: F_(1,59)_ = 11.403, *p* = 0.001, ƞ^2^ = 0.162] and CD16+ NK effector cells [106.54 ± 98.05 cells/ µ L vs. 43.66 ± 52.59 cells/μL. ANOVA: F_(1,59)_ = 13.600, *p* < 0.001, ƞ^2^ = 0.187] compared to healthy women (Table 4). There were no statistically significant differences in CD16− NK regulatory cells between groups, although there was a tendency for higher numbers among survivors [20.73 ± 13.35 cells/μL vs. 17.71 ± 13.60 cells/μL. ANOVA: F_(1,59)_ = 1.840, *p* = 0.180, ƞ^2^ = 0.030]. Breast cancer patients tended to have NK cell and NK cell subset counts that were closer to the counts exhibited by survivors and higher than healthy women.

### 3.8 CMV serostatus and CMV-specific IgG was similar among breast cancer survivors and healthy women

A similar proportion of survivors (44.4%) and healthy women (44.7%) were CMV+ (ANOVA: F_(1,63)_ = 0.001, *p* = 0.982, ƞ^2^ < 0.001) (Table 1). Within CMV+ participants, CMV-specific IgG was higher among survivors (*n* = 12; 17.28 ± 5.37 IU/mL) compared to healthy women (*n* = 17; 14.79 ± 6.44 IU/mL) but this was not statistically significant (*p* = 0.265). 80% of breast cancer patients were infected with CMV and CMV-specific IgG within CMV+ patients was lower than healthy women and survivors (*n* = 5; 13.81 ± 10.11 IU/mL).

### 3.9 Age, cardiorespiratory fitness, body composition and CMV serostatus did not impact total leukocyte counts, but influenced the characteristics of T cells, B cells and NK cells among breast cancer survivors and healthy women

Compared to healthy women, breast cancer survivors were older and shorter, exhibited lower cardiorespiratory fitness ( $\dot{V}O_{2}$ max), lower lean muscle mass, and had a greater percentage body fat and fat mass index [Summary of ANOVAs: F_(1,63)_ > 4.753, *p* < 0.033, ƞ^2^ > 0.070] (Table 1). As many of these variables influence blood immune profiles, ANCOVA analyses examined whether these variables were significant covariates, or significantly moderated the differences in immune cell status between groups. Although CMV serostatus was not different between groups, this variable was also included in ANCOVA analyses given the very strong influence CMV has on immune profiles (Chidrawar et al., 2009). Overall, these variables had no effect on leukocyte, lymphocyte, monocyte and neutrophil counts which remained similar between groups after adjustment for age, $\dot{V}O_{2}$ max, body fat percentage, lean mass, fat mass index and CMV serostatus [Summary of ANCOVAs: F_(1,62)_ < 2.668, *p* > 0.107, ƞ^2^ < 0.041; Supplementary Table S2].

#### 3.9.1 T cell profile

The statistically significant difference in CD4+ CM T cell count, which was higher among survivors compared to healthy women [ANOVA: F_(1,63)_ = 5.039, *p* = 0.028, ƞ^2^ = 0.074] was lost when controlling for age and $\dot{V}O_{2}$ max [Summary of ANCOVAs: F_(1,62)_ < 3.503 *p* > 0.066 ƞ^2^ < 0.053] but it was maintained when controlling for body fat percentage, lean mass, fat mass index and CMV status [Summary of ANCOVAs: F_(1,62)_ > 4.008 *p* < 0.050 ƞ^2^ > 0.061] (Supplementary Table S3). The statistically significant difference in CD8+NA T cell count, which was lower among survivors compared to healthy women (ANOVA: F_(1,63)_ = 4.690, *p* = 0.034, ƞ^2^ = 0.069) was lost when controlling for age, $\dot{V}O_{2}$ max, body fat percentage or fat mass index [Summary of ANCOVAs: F_(1,62)_ < 3.793, *p* > 0.056, ƞ^2^ < 0.058] but it was maintained when controlling for lean mass or CMV status [Summary of ANCOVAs: F_(1,62)_ > 4.147, *p* < 0.046, ƞ^2^ > 0.063] (Supplementary Table S3).

#### 3.9.2 T cell activation

Supplementary Table S4 shows ANCOVA analyses for CD4+ and CD8+ T cell activation. The statistically significant difference in the proportion of HLA-DR+ CD4+ CM T cells which was higher among survivors compared to healthy women [ANOVA: F_(1,57)_ = 4.160, *p* = 0.046, ƞ^2^ = 0.068] was lost when controlling for age, lean mass and fat mass index [Summary of ANCOVAs: F_(1,56)_ < 3.642 *p* > 0.061 ƞ^2^ < 0.061] but it was maintained when controlling for $\dot{V}O_{2}$ max, body fat percentage and CMV status [Summary of ANCOVAs: F_(1,56)_ > 4.211, *p* < 0.045, ƞ^2^ > 0.070]. The statistically significantly difference in the proportion of HLA-DR+ CD4+ EM and EMRA T cells which was higher among survivors [Summary of ANOVAs: F_(1,57)_ > 12.890, *p* < 0.001, ƞ^2^ > 0.184] was maintained when controlling for all variables [Summary of ANCOVAs: F_(1,56)_ > 9.295, *p* < 0.004, ƞ^2^ > 0.142].

For CD8+ T cells, the statistically significant difference in the proportion of HLA-DR+ CD8+ T cells, which was higher among survivors compared to healthy women [ANOVA: F_(1,57)_ = 6.408, *p* = 0.014, ƞ^2^ = 0.101], was lost when controlling for age and fat mass index [Summary of ANCOVAs: F_(1,56)_ < 3.168 *p* > 0.081 ƞ^2^ < 0.054] but it was maintained when controlling for $\dot{V}O_{2}$ max, body fat percentage, lean mass and CMV status [Summary of ANCOVAs: F_(1,56)_ > 4.162 *p* < 0.046 ƞ^2^ > 0.069]. The statistically significant difference in the proportion of HLA-DR+ CD8+ EM T cells, which was higher among survivors [ANOVA: F_(1,57)_ = 9.965, *p* = 0.003, ƞ^2^ = 0.149] was maintained when controlling all variables [Summary of ANCOVAs: F_(1,56)_ > 6.556 *p* < 0.013 ƞ^2^ > 0.105]. Finally, the statistically significant difference in the proportion of CD8+ EMRA T cells which was higher among survivors [ANOVA: F_(1,57)_ = 4.131, *p* = 0.047, ƞ^2^ = 0.068] was lost when controlling for all variables [Summary of ANCOVAs: F_(1,56)_ < 3.688 *p* > 0.060 ƞ^2^ < 0.062] except for body fat percentage and CMV status [Summary of ANCOVAs: F_(1,56)_ > 4.022 *p* < 0.050 ƞ^2^ > 0.067].

#### 3.9.3 CD4+/CD8+ ratio

Although the higher CD4+/CD8+ ratio among survivors compared to healthy women did not reach statistical significance [ANOVA: F_(1,63)_ = 2.945 *p* = 0.091 ƞ^2^ = 0.045], controlling for CMV serostatus strengthened the relationship [ANCOVA: F_(1,62)_ = 3.293 *p* = 0.074 ƞ^2^ = 0.050]. The CD4+/CD8+ ratio was not influenced by other variables [Summary of ANCOVAs: F_(1,62)_ < 2.663 *p* > 0.108 ƞ^2^ < 0.041) (Supplementary Table S2).

#### 3.9.4 TSCMs

There were no differences in the counts of CD4+ and CD8+ TSCMs between survivors and healthy women [ANOVA: CD4+ TSCMs: F_(1,63)_ = 0.473 *p* = 0.494 ƞ^2^ = 0.007. CD8+ TSCMs: F_(1,63)_ = 3.531 *p* = 0.065 ƞ^2^ = 0.053] and there were no effects of controlling for other variables [Summary of ANCOVAs: CD4+ TSCMs: F_(1,62)_ < 1.414 *p* > 0.239 ƞ^2^ < 0.022; CD8+ TSCMs: F_(1,62)_ < 3.687 *p* > 0.059 ƞ^2^ < 0.056] (Supplementary Table S5). Although the counts of CD8+ TSCMs among survivors compared to healthy women did not reach statistical significance, controlling for CMV serostatus strengthened the relationship [ANCOVA: F_(1,62)_ = 3.687 *p* = 0.059 ƞ2 = 0.056].

#### 3.9.5 B cells

The statistically significant difference in immature B cells which were higher among survivors compared to healthy women [ANOVA: F_(1,59)_ = 4.417 *p* = 0.040 ƞ^2^ = 0.070] was lost when controlling for age and fat mass index [Summary of ANCOVAs: F_(1,58)_ < 3.148 *p* > 0.081 ƞ^2^ < 0.051] but it was maintained when controlling for $\dot{V}O_{2}$ max, body fat, lean mass and CMV status [Summary of ANCOVAs: F_(1,58)_ > 4.457 *p* < 0.039 ƞ^2^ > 0.071] (Supplementary Table S5). There were no differences between groups in all other B cell subtypes [Summary of ANOVAs: F_(1,59)_ < 0.133 *p* > 0.717 ƞ^2^ < 0.002] and these patterns were maintained when controlling for all variables [Summary of ANCOVAs: F_(1,58)_ < 0.521 *p* > 0.473 ƞ^2^ < 0.009].

#### 3.9.6 NK cells

The statistically significant difference in NK cells and Effector NK cells which were higher among survivors compared to healthy women [Summary of ANOVAs: F_(1,59)_ > 11.403 *p* < 0.001 ƞ^2^ > 0.162] was maintained when controlling all variables [Summary of ANCOVAs: F_(1,58)_ > 8.272 *p* < 0.006 ƞ^2^ > 0.125] (Supplementary Table S5). There were no differences between groups in NK regulatory cells [ANOVA: F_(1,59)_ = 1.840 *p* = 0.180 ƞ^2^ = 0.030] and this pattern was maintained when controlling for all variables [Summary ANCOVAs: F_(1,58)_ < 2.115 *p* > 0.151 ƞ^2^ < 0.035].

### 3.10 Fat mass index was positively correlated with activation of CD4+ and CD8+ effector memory T cells

**FIGURE 3:**
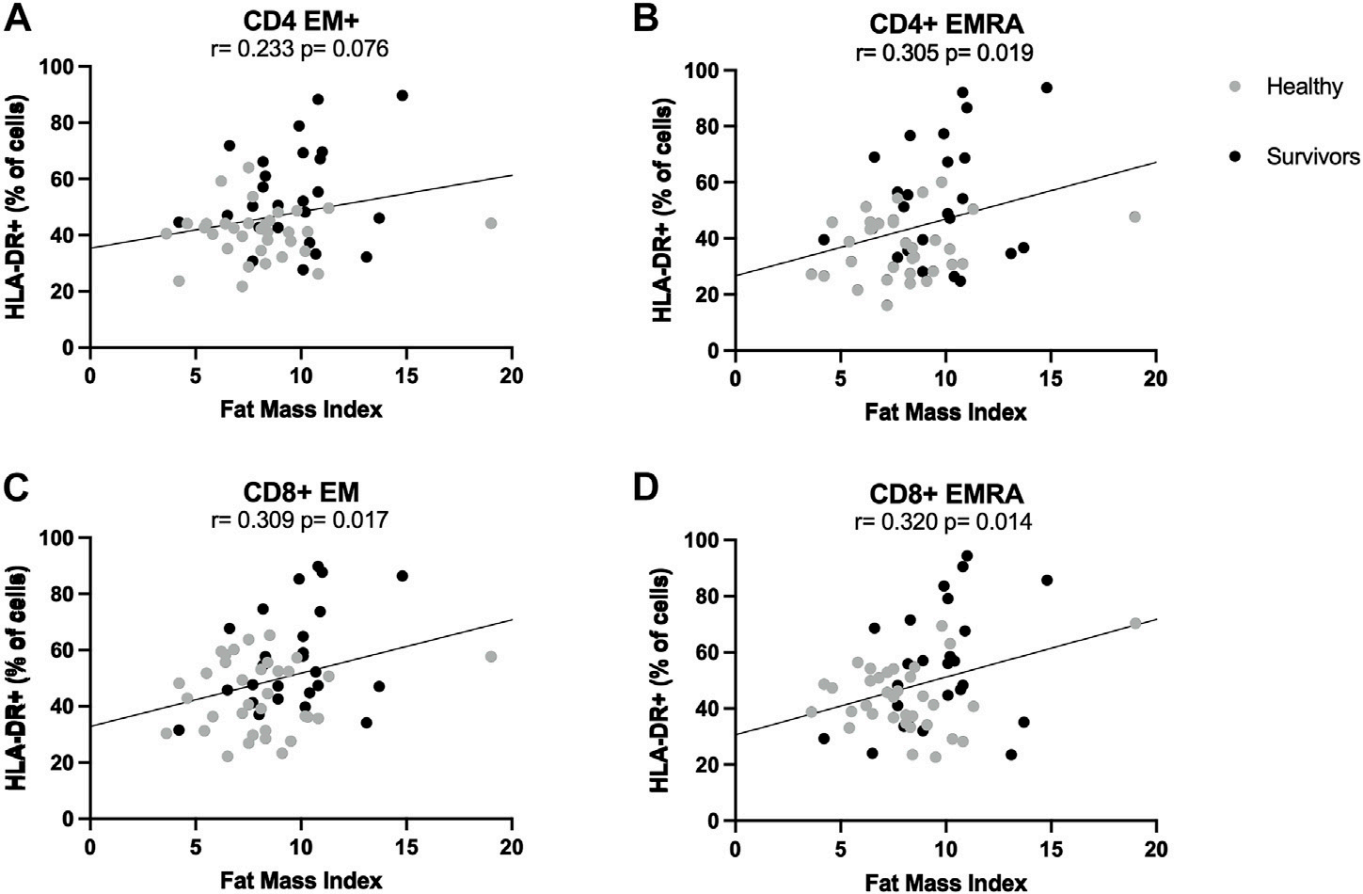
Correlations between T cell activation and fat mass index. Correlations between fat mass index and activation levels of CD4+ **(A,B)** and CD8+ **(C,D)** effector memory subsets. Activation was expressed as percentage of cells expressing HLA-DR. Both healthy women (*n* = 34) and breast cancer survivors (*n* = 25) were included in analyses (total *n* = 34 + 25 = 59). Statistical significance was considered at *p* < 0.05.

ANCOVA analyses showed that the statistically significant difference in the activation of CD4+ and CD8+ effector memory T cells between groups, which was higher among survivors compared to healthy women, was consistently and strongly influenced by fat mass index. Indeed, differences between groups were abolished when controlling for fat mass index, suggesting this variable could be driving the initial observations. Pearson correlations showed that across both groups, the proportion of HLA-DR+ CD4+ EMRA, CD8+ EM and CD8+ EMRA cells was positively correlated with fat mass index (Summary of Pearson correlations: *r* > 0.305, *p* < 0.019) (Figures 3A–D). In general, when correlations were examined for each group separately, there were no statistically significant relationships.

### 3.11 The effect of other participant characteristics on the positive association between fat mass index and activation of CD4+ and CD8+ effector memory cells

Regression analyses showed that the positive association between fat mass index and the percentage of both HLA-DR+ CD4+ EMRA and HLA-DR+ CD8+ EM (Summary of Pearsons correlations: *r* > 0.305, *p* < 0.019) was lost when controlling for $\dot{V}O_{2}$ max [adjusted linear regression: F_(1,56)_ < 3.957, *p* > 0.052, R < 0.310, R^2^ < 0.096 R^2^_Adjusted_ < 0.064 R^2^_Change_ < 0.064], but was maintained when controlling for age, lean mass and CMV status [adjusted linear regression: F_(1,56)_ > 4.114 *p* < 0.047 R > 0.305, R^2^ > 0.093 R^2^_Adjusted_ > 0.060 R^2^_Change_ > 0.066] (Supplementary Table S6). The positive correlation between the percentage of HLA-DR+ CD8+ EMRA and fat mass index (Pearsons correlations *r* = 0.320, *p* = 0.014) was maintained when controlling for all variables [adjusted linear regression: F_(1,56)_ > 4.622 *p* < 0.036 R > 0.320 R^2^ > 0.102 R^2^_Adjusted_ > 0.070 R^2^_Change_ > 0.073]. Finally, although there was no significant correlation between HLA-DR+ CD4+EM and fat mass index (*r* = 0.233, *p* = 0.076), a statistically significant relationship was shown when controlling for lean mass [adjusted linear regression: F_(1,56)_ = 4.594 *p* = 0.036 R = 0.316 R^2^ = 0.100 R^2^_Adjusted_ = 0.067 R^2^_Change_ = 0.074].

## 4 Discussion

This study investigated whether breast cancer survivors exhibit a different leukocyte profile in blood compared to healthy women. Uniquely, this study also included a comprehensive analysis of participant characteristics that influence immune profiles, including age, CMV serostatus, cardiorespiratory fitness and body composition. In summary, this study has shown that, although total leukocyte counts were not different between groups, statistically significant differences were found when examining further leukocyte subtypes. Survivors exhibited higher levels of CD4+ central memory T cells, lower levels of CD8+ naïve T cells and a greater proportion of activated CD4+ and CD8+ effector-memory T cells. B cells and their subtypes also tended to be higher among survivors, but only immature B cells showed a statistically significant difference between groups. Furthermore, total NK cells and CD16+ effector cells were significantly higher among survivors. Analysis of covariance revealed divergent moderating effects of age, CMV serostatus, cardiorespiratory fitness and body composition on the differences in immune cell status between groups, depending on the cell type examined. Most consistently, across all participants, fat mass index was positively associated with the proportion of activated CD4+ EMRA and CD8+ EM/EMRA T cells. For CD8+ EMRA cells, this association withstood statistical adjustment for all variables, including age, CMV serostatus, lean mass and cardiorespiratory fitness.

An important finding from this study is that compared to healthy women, breast cancer survivors within 2 years of treatment, exhibited a greater proportion of HLA-DR+ activated CD4+ and CD8+ effector memory cells. In addition, our analyses of HLA-DR cell surface expression levels indicate that–on a per cell basis–the magnitude of activation was also greater among survivors compared to healthy women (data not shown). High T cell activation 6–12 months after treatment has been reported by analysis of serial blood samples from patients with brain cancer, sarcoma, Non-Hodgkin lymphoma and breast cancer, but has not been compared to healthy women (Mackall et al., 1994; Hakim et al., 1997). We show that the proportion of activated CD4+ and CD8+ T cells overall was +Δ31% higher among survivors compared to healthy women. T cell activation is important for a targeted immune response and an absence, deficiency, and/or a lack of co-stimulation can result in anergy, favouring tumour escape (Swann and Smyth, 2007). Activation of T cells in blood, and in the tumour, has been positively associated with long-term disease-free survival in a variety of cancers (McCracken et al., 2015). For example, in breast cancer, patients who responded better to neoadjuvant chemotherapy had significantly higher levels of HLA-DR expression among CD8+ T cells that had infiltrated the tumour, compared to non-responders (Saraiva et al., 2018). The importance of T cell activation is emphasised by immunotherapies that block inhibitory molecules (e.g., anti CTLA-4 or anti PD-1/PDL-1 therapies) (Seidel et al., 2018) or immunotherapies that infuse activated antigen-specific T cells (Saibil and Ohashi, 2020). Thus, T cell activation among breast cancer survivors in the present study might reflect their positive response to treatment and might represent cancer-specific immunity, which has previously been shown to positively correlate with survival in breast cancer (Bailur et al., 2015). Although we did not assess cancer-specific immunity in the present study, we have previously shown in a sub-group (*n* = 10) of the breast cancer survivors included, that their T cells recognize tumour-associated antigens (e.g., mammaglobin) (Arana Echarri et al., 2023).

Other data from this study could also be interpreted as reflecting a previous anti-tumour response. For example, the lower CD8+ naïve T cell counts and higher CD4+ central memory cell counts among breast cancer survivors, might reflect a previous expansion of effector cells and concomitant shrinking of the naïve T cell pool. These changes are typically followed by a subsequent contraction of effector cells, and maintenance of central memory cells, to provide long-lived memory once tumour cells have been eliminated (Farber et al., 2014). Indeed, these central memory cells might provide continued surveillance and could expand upon antigen re-exposure with tumour recurrence. Among T cells, increasing emphasis has been placed on stem cell like memory T cells (TSCMs), due to their self-renewal, multipotency, and anti-tumour properties (Gattinoni et al., 2011). TSCMs differentiate from the naïve T cell pool and their accumulation is facilitated by IL-7 and IL-15 (Cieri et al., 2013). In the present study however, there were no statistically significant differences between groups in the number of CD4+ or CD8+ TSCMs, but cell numbers among survivors were approximately half of those exhibited by healthy women. It is possible that statistically significant differences between groups might have been observed closer to the time of treatment, or with a different identification strategy. For example, in the present study, TSCMs were robustly identified using CD127 and CD95 in combination with CD27 and CD45RA (Gattinoni et al., 2011; Schmueck-Henneresse et al., 2015; Curran et al., 2019). However, other strategies identify naïve cells using CD45RO, CCR7, CD62L and subsequently use CD95 in isolation (Lugli et al., 2013).

Furthermore, the present study found a trend for higher counts of total B cells and their subtypes among breast cancer survivors, whereas immature B cells were significantly higher than in healthy women. There is controversy over whether B cells contribute to pro- or anti-tumour responses, and this relationship is influenced by the frequencies of functionally distinct B cell subtypes (Fremd et al., 2013; Tsou et al., 2016). Experiments in mice with melanoma have shown that depleting total B cells impaired CD4+ and CD8+ effector memory T cell proliferation following tumour antigen exposure, doubling tumour volume and metastasis (Dilillo et al., 2010). Similar results have been shown in mouse models of glioblastoma (Candolfi et al., 2011). In human studies, it has been shown that non-progressing patients with metastatic melanoma, lung adenocarcinoma, or renal cancer have elevated plasmablasts in blood and functional anti-tumour antibody production (Defalco et al., 2018).

Although one interpretation of differences in leukocyte sub-types among breast cancer survivors compared to healthy women relates to a potentially favourable immune response to cancer, another interpretation could be that the observations made in this study reflect immunosenescence (Makinodan and Kay, 1980). Hallmarks of immunosenescence include an age-associated decline in naïve T cells and an accumulation of memory T cells, due to thymic involution, changes to haematopoietic stem cells, and chronic exposure to immunodominant antigens (Franceschi et al., 2000; Chinn et al., 2012). In the present study, we show that breast cancer survivors (56 ± 6 years) exhibited higher levels of CD4+ central memory T cells and lower levels of CD8+ Naïve T cells compared to healthy women (45 ± 11 years). In addition, survivors exhibited higher activation levels of CD4+ and CD8+ effector-memory T cells than healthy women, which potentially contribute to inflammageing (Macaulay et al., 2013). In support, the higher immature B cell count, higher CD4+ central memory T cell count, and lower CD8+ naive T cell count among breast cancer survivors, which were significantly different between groups, were lost when controlling for age, but–importantly (see next paragraph)—were not lost when controlling for CMV serostatus. These observations could be a combined effect of the statistically significant age difference between groups, and the exposure of survivors to tumour antigens and cancer therapy. Indeed, cytotoxic drugs can induce apoptosis of leukocyte populations, and in particular hematopoietic stem cells, leading to a fall in cell reserves which also have impaired self-renewal properties, therefore giving rise to long-term bone marrow injury (Testa et al., 1985; Shao et al., 2013; Rébé and Ghiringhelli, 2015; Kinsella and Dudakov, 2020; Truong et al., 2021). In addition, cytotoxic drugs cause senescence in the thymic stromal compartment, characterised by release of inflammatory molecules, depletion of thymocytes and other tissue-resident cells important for T cell development and maturation (e.g., thymic epithelial cells), leading to a reduction of the T cell repertoire (Kinsella and Dudakov, 2020).

It is well established that two strong drivers of immunosenescence are age and CMV infection. Initial analyses in the present study identified differences in the counts and activation levels of several cell types when comparing breast cancer survivors to healthy women. Further analyses were undertaken to understand whether age and CMV contribute towards these group differences. In summary, the counts of CD4+ CM T cells were higher and CD8+ NA T cells lower among breast cancer survivors. However, this statistically significant difference was lost when controlling for age but maintained when controlling for CMV serostatus. Likewise, the greater proportion of activated CD4+ CM T cells, total CD8+ and CD8+ EMRA T cells among survivors was also lost when controlling or age, but not CMV serostatus. These findings imply that age may have a stronger influence on the characteristics of the T cell pool than CMV among breast cancer survivors. Thus, the influence that cancer and its treatment might have on the T cell pool, could be interpreted as being less dramatic than the effects of age. However, for other measurements, including the proportion of activated CD4+ EM and EMRA and CD8+ EM T cells, which were higher among survivors than healthy women, remained statistically significant when controlling for both age and CMV (and all other participant characteristics). Thus, an interpretation of these latter findings is that cancer treatment might have a stronger influence than age and CMV on some T cell sub-types compared to others. In support, our preliminary analysis of recently diagnosed patients (*n* = 5) who had not undergone treatment, show that activation of CD4+ and CD8+ EM and EMRA T cells was closer to the levels exhibited by healthy women.

Another commonly assessed marker of immunosenescence is the ratio of CD4+ T cells to CD8+ T cells (Ferguson et al., 1995; Strindhall et al., 2013; Tylutka et al., 2021). A normal ratio is considered to be 1.0 to 2.5 (McBride and Striker, 2017), but can be influenced by age, sex, genetics and infections (Amadori et al., 1995; Wikby et al., 2008). A low ratio (<1.0) is a hallmark of immunosenescence and was included in the “Immune Risk Profile” (Ferguson et al., 1995; Strindhall et al., 2013; Tylutka et al., 2021) but high ratios (>2.5) have also been reported among older people (Tylutka et al., 2020), and have been linked with autoimmune disease (Daskalova et al., 1997), frailty and shorter survival (Pawelec, 2019). In the present study, the CD4+/CD8+ ratio was higher among survivors (3.7 ± 2.7) compared to healthy women (2.8 ± 1.5) but this difference did not reach statistical significance (*p* = 0.091). However, the percentage of survivors with an inverted (<1.0) ratio or higher ratio (>2.5) was greater than among healthy women (survivors: 66.7% vs. healthy women: 47.3%; *p* = 0.14), which could be a combined effect of cancer and inflammation (Singh et al., 2019). Age and CMV appeared not to robustly impact the CD4+/CD8+ ratio.

In addition to age and CMV, the present study extended our analyses of participant characteristics that might influence leukocyte subset characteristics in blood. For example, the higher count of CD4+ CM T cells and lower count of CD8+ NA T cells that was exhibited by survivors compared to healthy women was no longer statistically significant when controlling for cardiorespiratory fitness. Indeed, immune competency in general is known to be positively associated with cardiorespiratory fitness (Nehlsen-Cannarella et al., 1991; Emery et al., 2022) and the effects on the T cell pool are considered to be independent of CMV (Spielmann et al., 2011). However, these relationships have not been shown in cancer settings until now. Despite the effects on some cell counts, cardiorespiratory fitness did not influence the difference between groups in the proportion of HLA-DR+ CD4+ CM, EM, EMRA and HLA-DR+ total CD8+ and CD8+ EM T cells, which after statistical adjustment, remained significantly higher among survivors compared to healthy women. The finding that T cell activation appeared not to be affected by cardiorespiratory fitness among breast cancer survivors was consistent when HLA-DR cell surface expression levels–indicating the magnitude of activation on a per cell basis–was examined.

Among our measurements of body composition, the most consistent observations were made with fat mass index. When controlling for this variable, the higher count of CD8+ NA T cells and the greater proportion of HLA-DR+ CD4+ CM, total CD8+ T cells and CD8+ EMRA cells among breast cancer survivors compared to healthy women was no longer statistically significant. Subsequent analyses showed that across all participants, there was a positive correlation between fat mass index and the proportion of HLA-DR+ CD4+ EMRA and CD8+ EM. These relationships were maintained when controlling for age, lean mass and CMV serostatus, but lost when controlling for cardiorespiratory fitness. The only cell sub-type for which the proportion of HLA-DR+ positive cells remained positively correlated with fat mass index across groups after statistical adjustment for all variables was CD8+ EMRA T cells. This finding shows a potentially important relationship between adiposity and a subset of T cells that could contribute to inflammation and immune dysfunction in overweight and obesity. In support, a recent study has also shown a positive association between CD8+ EMRA T cell activation and body fat among older adults (Boßlau et al., 2021). Indeed, in non-cancer settings, overweight and obesity have been linked with overall dysfunction of the immune system (Marti et al., 2001). A higher body mass index has been associated with higher counts of total leukocytes, total lymphocytes, CD4+ T cells, regulatory T cells (Van Der Weerd et al., 2012) and effector memory αβ T cells (Alam et al., 2012), most likely reflecting obesity-induced inflammation (Womack et al., 2007; Ilavská et al., 2012). However, very few studies have examined the effect of body composition on leukocyte sub-types in cancer settings, except for mostly γδ T cells or NK cells (Donninelli et al., 2017; Viel et al., 2017; Michelet et al., 2018; Bähr et al., 2020; Campiotti et al., 2021; Elaraby et al., 2021). It is likely that obesity-associated changes to leukocyte profiles and function are driven by cross-talk between adipose tissue, bone marrow and the thymus, as well as direct effects that adipokines can have on immune cells (Dixit, 2008).

An additional novelty of the present study is that five women who had been diagnosed with breast cancer, but had not yet received any form of treatment, were included as part of a preliminary qualitative analysis. Patients were characterised by: 1) higher counts of all leukocytes compared to both healthy women and survivors, 2) similar T cell profile and T cell subset activation patterns to both groups, 3) similar counts of CD8+ NA T cells to both groups, 4) similar counts of CD4+ CM cells to survivors but higher counts than healthy women, 5) lower proportion of activated CD4+ and CD8+ T cells and subsets than survivors but an activation profile more closely aligned with healthy women, 6) lower CD4+ TSCM counts compared to both groups, 7) higher CD8+ TSCM counts compared to survivors but similar to healthy women, 8) similar total B/NK cell and subset counts to survivors but higher than healthy women and 9) the percentage of patients with an inverted or higher CD4+/CD8+ ratio (60%) was higher than healthy women (47.3%). These preliminary qualitative observations indicate that when immune profiles are assessed prior to treatment, the counts and distribution of cell types are generally aligned with breast cancer survivors (i.e., implying there could be a disease-associated phenotype irrespective of treatment). However, there was large inter-individual variation among patients which could influence prognosis and/or response to treatment. In support, studies have linked baseline pre-treatment levels of some immune cells in blood with clinical outcomes among patients with breast cancer. For example, higher blood lymphocyte counts have been linked with pathological complete responses after neoadjuvant chemotherapy in primary breast cancer (Qian et al., 2018), and with longer overall survival and disease-free survival in triple negative breast cancer (He et al., 2016). While other studies have reported higher counts of effector memory T cells and lower counts of naïve T cells in early stage breast cancer patients (Poschke et al., 2012), the prognostic and predictive value of these measurements remains unknown. It is recognised that a future research priority is to identify immune profiles that predict clinical outcomes in breast cancer to support personalised treatment and aid decision making (Batalha et al., 2021).

In this manuscript, breast cancer survivors and healthy women were recruited as part of separate studies and some inclusion criteria were different. For example, there was a narrower age range for breast cancer survivors (*n* = 27, 35–69 years) compared to healthy women (*n* = 38, 25–69 years). Statistical analyses conducted after removing *n* = 10 healthy women under the age of 35 years are presented in the Supplementary Table S7, and nearly all differences between groups remain largely the same. In addition, because breast cancer survivors were recruited as part of an exercise training study, these women were required to self-report that they did not undertake regular structured physical activity for more than 30 min on two or more occasions per week, whereas among healthy women, physical activity level was not a discriminating factor for study entry. Participants were further characterized using the international physical activity questionnaire. Between groups, there were no statistically significant differences in self-reported time engaged in light or moderate intensity physical activity. However, time engaged in vigorous activities were higher (Supplementary Table S7) reflected by a higher $\dot{V}O_{2}$ max among healthy women, used in our statistical analyses to examine the influence of an active lifestyle. The characteristics of breast cancer survivors are largely representative of this population and the differences between groups are an important feature of this work. Although previous studies have compared immunological variables between people living with or beyond cancer to healthy controls–sometimes matched for age or BMI–no studies have systematically examined the influence of such a broad range of participant characteristics, and dataset heterogeneity is critical to understand these relationships. Finally, it should be considered that the influence of menopausal status could not be investigated. All breast cancer survivors were post-menopausal, and even if some women were pre-menopausal prior to treatment, menopause would have been induced during treatment. Among healthy women only, cell counts and activation status were not different between *n* = 24 pre-menopausal and *n* = 14 post-menopausal women, except for very slightly higher plasmablasts and immature B cells among pre-menopausal women (data not shown).

In conclusion, this study has shown that compared to healthy women, breast cancer survivors exhibited higher levels of CD4+ central memory T cells, lower levels of CD8+ naïve T cells and higher activation levels of CD4+ and CD8+ effector-memory T cells. Immature B cells, total NK cells and CD16+ effector cells were also significantly higher among survivors. Uniquely, this study also shows that fat mass index is positively associated with the activation levels of CD4+ and CD8+ effector memory T cells across healthy women and breast cancer survivors. Further, the relationship between fat mass index and CD8+ EMRA T cell counts withstood statistical adjustment for all variables, including age, CMV serostatus, cardiorespiratory fitness and other measures of body composition, such as lean mass. This finding shows a potentially important relationship between adiposity and a subset of T cells that could contribute to inflammation and immune dysfunction in overweight and obesity. Overall, our results emphasise that age, CMV serostatus, cardiorespiratory fitness and in particular, body composition, should be taken into consideration, when profiling leukocytes in blood in humans. Future studies may reveal similar associations with other participant characteristics, such a habitual physical activity level and diet.

## Data Availability

The raw data supporting the conclusion of this article will be made available by the authors, without undue reservation.
