# Supplementary material for: Immune cell status, cardiorespiratory fitness and body composition among breast cancer survivors and healthy women: a cross sectional study
Source: Front Physiol. 2023 Jun 1;14:1107070. doi: 10.3389/fphys.2023.1107070 (PMC10267418; doi:10.3389/fphys.2023.1107070)
Supplement: Supplementary file 1 [file DataSheet1.docx]

**Supplementary Tables**

**S. Table 1. Diagnostic and treatment information for breast cancer survivors and patients (extended)**

| **Survivor** | **Diagnosis** | | **Months since diagnosis** | **Months since surgery** | **Treatment** | | | |
| --- | --- | --- | --- | --- | --- | --- | --- | --- |
|  |  |  |  |  | **Surgery** | **Chemotherapy** | **Radiotherapy** | **Endocrine therapy** |
| 1 | G3 T2 N1 ER/PR+ HER2+ | | 19 | 17 | Lumpectomy | FEC-TH (adj) | yes | Type 1 |
| 2 | G3 T1 N0 multifocal ER/PR- HER2+ | | 11 | 8 | Mastectomy | FEC-TH | - | - |
| 3 | G2 T1 N0 ductal ER/PR+ HER2- | | 19 | 15 | Lumpectomy | - | yes | Type 1 |
| 4 | DCIS (Tis N0) ER/PR+ HER2- | | 15 | 14 | Lumpectomy | - | yes | - |
| 5 | G3 T1 N1 ER/PR+ HER2- | | 14 | 13 | Lumpectomy | - | yes | Type 2 |
| 6 | G2 T2 N0 ER/PR+ HER2- | | 7 | 6 | Lumpectomy | - | yes | Type 3 |
| 7 | G2 T1 N0 multifocal (+DCIS) ER/PR+ HER2- | | 13 | 11 | Mastectomy | FEC-T | - | Type 2 |
| 8 | G2 T2 N0 ER/PR+ HER2- | | 10 | 8 | Lumpectomy | - | yes | Type 2 |
| 9 | G2 T2 N0 ER/PR+ HER2- | | 23 | 10 | Mastectomy | FEC-T | yes | Type 2 |
| 10 | G1 T1 N0 ER/PR+ HER2- | | 8 | 7 | Lumpectomy | - | yes | - |
| 11 | G3 T1 N1 ER/PR+ HER2- | | 8 | 6 | Lumpectomy | - | yes | Type 1 |
| 12 | G3 T2 N1 ER/PR- HER2- | | 12 | 11 | Mastectomy | FEC-T | yes | - |
| 13 | DCIS (Tis N0) | | 9 | 8 | Lumpectomy | - | yes | - |
| 14 | G2 T2 N1 ER/PR+ HER2- | | 15 | 14 | Mastectomy | FEC-T | yes | Type 3 |
| 15 | DCIS (Tis N0) * | | 20 | 19 | Mastectomy | - | - | - |
| 16 | DCIS (Tis N0) | | 22 | 21 | Lumpectomy | - | yes | - |
| 17 | G2 T1 N0 ER/PR+ HER2- | | 26 | 25 | Lumpectomy | - | yes | Type 3 |
| 18 | DCIS (Tis N0) ER/PR+ HER2- | | 14 | 9 | Mastectomy | - | - | Type 1 |
| 19 | G2 T1 N0 ER/PR+ HER2- | | 10 | 10 | Lumpectomy | - | yes | Type 2 |
| 20 | G2 T3 N3 ER/PR+ HER2- | | 29 | 23 | Mastectomy | FEC-T (neoadj) | yes | Type 3 |
| 21 | G2 T1 N2 (with DCIS) ER/PR+ HER2- | | 9 | 6 | Mastectomy | - | yes | Type 1b |
| 22 | G2 T2 N0 ER/PR+ HER2- | | 7 | 5 | Lumpectomy | - | yes | Type 1 |
| 23 | G3 T2 N0 ER/PR- HER2- | | 7 | 7 | Lumpectomy | - | yes | - |
| 24 | G3 T1 N1 ER/PR+ HER2- | | 10 | 9 | Lumpectomy | FEC-T | yes | Type 2 |
| 25 | DCIS (Tis N0) | | 19 | 16 | Mastectomy | - | - | - |
| 26 | G2 T1 N0 ER/PR+ HER2- | | 7 | 5 | Lumpectomy | - | yes | Type 1 |
| 27 | G2 T3 N2 ER/PR+ HER2- | | 16 | 15 | Mastectomy | AC-Taxol | yes | Type 3 |
| **Patient** | | **Diagnosis** | | | **Treatment** | | | |
| 1 | | G3 N1 ER- HER2- | | | Neoadjuvant FEC-T, lumpectomy | | | |
| 2 | | G3 N1 ER- HER2- | | | Neoadjuvant FEC-T, mastectomy | | | |
| 3 | | G3 N0 ER- HER2- | | | Neoadjuvant FEC-T, mastectomy | | | |
| 4 | | G2 N1 ER+ HER2- | | | Neoadjuvant FEC- paclitaxel, lumpectomy | | | |
| 5 | | G3 N0 ER+ HER2+ | | | Neoadjuvant FEC-TPH, lumpectomy | | | |

*G (grade) refers to the Histologic grade (G1, G2, G3, etc). T (tumour) and N (nodes) values refer to the TNM staging to define the characteristics of the tumour 0 to 4 (T) and 0 to 3 (N).* ***Other Abbreviations key:*** *adj= adjuvant, DCIS= Ductal Carcinoma In Situ, neoadj= neoadjuvant, RT= Radiotherapy.* ***Treatment key for Survivors:*** *FEC-T= Fluorouracil, Epirubicin and Cyclophosphamide for 3 cycles followed by Docetaxel for 3 cycles; FEC-TH= Fluorouracil, Epirubicin and Cyclophosphamide for 3 cycles followed by Docetaxel for 3 cycles, plus Herceptin. AC-taxol*= *Doxorubicin, cyclophosphamide and Paclitaxel* (*Taxol) weekly for 9-12 weeks.* *Type 1= Endocrine therapy regimen 1: letrozole (aromatase inhibitor), Type 1b= Endocrine therapy regimen 1b: letrozole (aromatase inhibitor) plus LHRHa, Type 2= Endocrine therapy regimen 2: anastrozole (aromatase inhibitor), Type 3= Endocrine therapy regimen 3: tamoxifen. *= This patient had a prior breast cancer diagnosis in 1995 but was treated elsewhere; no more detail is known about this previous diagnosis and/or treatment.* ***Treatment key for Patients:*** *FEC-T= Fluorouracil, Epirubicin and Cyclophosphamide for 3 cycles followed by Docetaxel for 3 cycles, FEC-paclitaxel= Fluorouracil, Epirubicin and Cyclophosphamide for 3 cycles followed by Paclitaxel for 3 cycles, FEC-TPH= FEC-T, but including Pertuzumab and Trastuzumab in cycles 4-6.*

**S. Table 2: Leukocyte counts after statistical** **adjustment for age, VO_2_ max, body fat (%), lean mass (kg), fat mass index or CMV status.**

| **Cells and covariates** | **Healthy** | **Survivors** | **Original significance** | **Adjusted univariate ANOVA** |
| --- | --- | --- | --- | --- |
| *Total leukocytes (cells* x 10^10^/L*)* | 5.27 ± 1.52 | 5.06 ± 1.33 | ns | F_(1,63)_=0.253 p=0.617 ƞ^2^=0.004 |
| Age adjusted |  |  | ns | F_(1,62)_=1.244 p=0.269 ƞ^2^=0.020 |
| VO_2_ max adjusted |  |  | ns | F_(1,62)_=0.035 p=0.851 ƞ^2^=0.001 |
| Body Fat (%) adjusted |  |  | ns | F_(1,62)_=0.117 p=0.734 ƞ^2^=0.002 |
| Lean mass adjusted |  |  | ns | F_(1,62)_=0.372 p=0.544 ƞ^2^=0.006 |
| Fat mass index adjusted |  |  | ns | F_(1,62)_=0.479 p=0.492 ƞ^2^=0.008 |
| CMV status adjusted |  |  | ns | F_(1,62)_=0.248 p=0.620 ƞ^2^=0.004 |
| *Lymphocytes (cells* x 10^10^/L*)* | 1.55 ± 0.48 | 1.46 ± 0.38 | ns | F_(1,63)_=0.630 p=0.430 ƞ^2^=0.01 |
| Age adjusted |  |  | ns | F_(1,62)_=2.668 p=0.107 ƞ^2^=0.041 |
| VO_2_ max adjusted |  |  | ns | F_(1,62)_=0.265 p=0.609 ƞ^2^=0.004 |
| Body Fat (%) adjusted |  |  | ns | F_(1,62)_=0.511 p=0.477 ƞ^2^=0.008 |
| Lean mass adjusted |  |  | ns | F_(1,62)_=0.384 p=0.538 ƞ^2^=0.006 |
| Fat mass index adjusted |  |  | ns | F_(1,62)_=1.131 p=0.292 ƞ^2^=0.018 |
| CMV status adjusted |  |  | ns | F_(1,62)_=0.602 p=0.441 ƞ^2^=0.010 |
| *Monocytes (cells* x 10^10^/L*)* | 0.41 ± 0.20 | 0.41± 0.18 | ns | F_(1,63)_<0.01 p=0.927 ƞ^2^<0.01 |
| Age adjusted |  |  | ns | F_(1,62)_=2.652 p=0.108 ƞ^2^=0.041 |
| VO_2_ max adjusted |  |  | ns | F_(1,62)_=0.076 p=0.784 ƞ^2^=0.001 |
| Body Fat (%) adjusted |  |  | ns | F_(1,62)_=0.000 p=0.997 ƞ^2^=0.000 |
| Lean mass adjusted |  |  | ns | F_(1,62)_=0.005 p=0.942 ƞ^2^<0.001 |
| Fat mass index adjusted |  |  | ns | F_(1,62)_=0.052 p=0.820 ƞ^2^=0.001 |
| CMV status adjusted |  |  | ns | F_(1,62)_=0.001 p=0.980 ƞ^2^=0.000 |
| *Neutrophils (cells* x 10^10^/L*)* | 3.31 ± 1.19 | 3.17 ± 1.13 | ns | F_(1,63)_=0.202 p=0.655 ƞ^2^<0.01 |
| Age adjusted |  |  | ns | F_(1,62)_=0.429 p=0.515 ƞ^2^=0.007 |
| VO_2_ max adjusted |  |  | ns | F_(1,62)_=0.014 p=0.905 ƞ^2^=0.000 |
| Body Fat (%) adjusted |  |  | ns | F_(1,62)_=0.070 p=0.793 ƞ^2^=0.001 |
| Lean mass adjusted |  |  | ns | F_(1,62)_=0.390 p=0.535 ƞ^2=^0.006 |
| Fat mass index adjusted |  |  | ns | F_(1,62)_=0.287 p=0.594 ƞ^2^=0.005 |
| CMV status adjusted |  |  | ns | F_(1,62)_=0.198 p=0.658 ƞ^2^=0.003 |
| *CD4+ T cells (cells/µL)* | 844.08 ± 282.90 | 873.96 ± 260.47 | ns | F_(1,63)_=0.340 p=0.562 ƞ^2^=0.005 |
| Age adjusted |  |  | ns | F_(1,62)_=0.177 p=0.676 ƞ^2^=0.003 |
| VO_2_ max adjusted |  |  | ns | F_(1,62)_=0.103 p=0.750 ƞ^2^=0.002 |
| Body Fat (%) adjusted |  |  | ns | F_(1,62)_=0.107 p=0.744 ƞ^2^=0.002 |
| Lean mass adjusted |  |  | ns | F_(1,62)_=0.558 p=0.458 ƞ^2^=0.009 |
| Fat mass index adjusted |  |  | ns | F_(1,62)_=0.067 p=0.797 ƞ^2^=0.001 |
| CMV status adjusted |  |  | ns | F_(1,62)_=0.334 p=0.565 ƞ^2^=0.005 |
| *CD8+ T cells (cells/µL)* | 356.50 ± 178.93 | 297.44 ± 129.20 | ns | F_(1,63)_=2.018 p=0.160 ƞ^2^=0.031 |
| Age adjusted |  |  | ns | F_(1,62)_=1.212 p=0.275 ƞ^2^=0.019 |
| VO_2_ max adjusted |  |  | ns | F_(1,62)_=0.693 p=0.408 ƞ^2^=0.011 |
| Body Fat (%) adjusted |  |  | ns | F_(1,62)_=1.345 p=0.251 ƞ^2^=0.021 |
| Lean mass adjusted |  |  | ns | F_(1,62)_=1.522 p=0.222 ƞ^2^=0.024 |
| Fat mass index adjusted |  |  | ns | F_(1,62)_=2.249 p=0.139 ƞ^2^=0.035 |
| CMV status adjusted |  |  | ns | F_(1,62)_=2.238 p=0.140 ƞ^2^=0.035 |
| *CD4/CD8 ratio* | 2.82 ± 1.47 | 3.73 ± 2.66 | ns | F_(1,63)_=2.945 p=0.091 ƞ^2^= 0.045 |
| Age adjusted |  |  | ns | F_(1,62)_=0.513 p=0.477 ƞ^2^=0.008 |
| VO_2_ max adjusted |  |  | ns | F_(1,62)_=0.995 p=0.322 ƞ^2^= 0.016 |
| Body Fat (%) adjusted |  |  | ns | F_(1,62)_=1.717 p=0.195 ƞ^2^=0.027 |
| Lean mass adjusted |  |  | ns | F_(1,62)_=2.663 p=0.108 ƞ^2^=0.041 |
| Fat mass index adjusted |  |  | ns | F_(1,62)_=2.419 p=0.125 ƞ^2^=0.038 |
| CMV status adjusted |  |  | ns | F_(1,62)_=3.293 p=0.074 ƞ^2^=0.050 |

*Healthy women (n=38) and breast cancer survivors (n=27) were compared. Data are mean ±* *standard deviation (SD).* ƞ^2^ =partial eta squared. “ns” = not statistically significant between groups in original ANOVA analysis. Statistical significance was considered to be p<0.05*. Abbreviations key: CMV= Cytomegalovirus, VO_2_ max= cardiorespiratory fitness.*

**S. Table 3. T cell subset counts after statistical adjustment for age, VO_2_ max, body fat (%), lean mass (kg), fat mass index or CMV status.**

| **Cells and covariates** | **Healthy** *(cells/µL)* | **Survivors (***cells/µL)* | **Original significance** | **Adjusted univariate ANOVA** |
| --- | --- | --- | --- | --- |
| *CD4+NA* | 408.11 ± 236.63 | 371.85 ± 208.50 | ns | F_(1,63)_=0.878 p=0.352 ƞ^2^=0.014 |
| Age adjusted |  |  | ns | F_(1,62)_=2.503 p=0.119 ƞ^2=^0.039 |
| VO_2_ max adjusted |  |  | ns | F_(1,62)_=1.187 p=0.280 ƞ^2=^0.019 |
| Body Fat (%) adjusted |  |  | ns | F_(1,62)_=1.239 p=0.270 ƞ^2^=0.020 |
| Lean mass adjusted |  |  | ns | F_(1,62)_=0.896 p=0.348 ƞ^2^=0.012 |
| Fat mass index adjusted |  |  | ns | F_(1,62)_=0.729 p=0.397 ƞ^2^=0.012 |
| CMV status adjusted |  |  | ns | F_(1,62)_=0.933 p=0.338 ƞ^2^=0.015 |
| *CD4+CM* | 345.55 ± 160.75 | **417.11 ± 109.48*** | p<0.05 | **F_(1,63)_=5.039 p=0.028 ƞ^2^=0.074** |
| Age adjusted |  |  | lost | F_(1,62)_=2.827 p=0.098 ƞ^2^=0.044 |
| VO_2_ max adjusted |  |  | lost | F_(1,62)_=3.503 p=0.066 ƞ^2^=0.053 |
| **Body Fat (%) adjusted** |  |  | **maintained** | **F_(1,62)_=4.329 p=0.042 ƞ^2^=0.065** |
| **Lean mass adjusted** |  |  | **maintained** | **F_(1,62)_=6.023 p=0.017 ƞ^2^=0.089** |
| **Fat mass index adjusted** |  |  | **maintained** | **F_(1,62)_=4.008 p=0.050 ƞ^2^=0.061** |
| **CMV status adjusted** |  |  | **maintained** | **F_(1,62)_=5.029 p=0.029 ƞ^2^=0.075** |
| *CD4+EM* | 65.29 ± 58.66 | 69.26 ± 38.39 | ns | F_(1,63)_=1.600 p=0.211 ƞ^2^=0.025 |
| Age adjusted |  |  | ns | F_(1,62)_=1.456 p=0.232 ƞ^2^=0.023 |
| VO_2_ max adjusted |  |  | ns | F_(1,62)_=1.666 p=0.202 ƞ^2^=0.026 |
| Body Fat (%) adjusted |  |  | ns | F_(1,62)_=1.749 p=0.191 ƞ^2^=0.027 |
| Lean mass adjusted |  |  | ns | F_(1,62)_=2.629 p=0.110 ƞ^2^=0.041 |
| Fat mass index adjusted |  |  | ns | F_(1,62)_=0.738 p=0.394 ƞ^2^=0.012 |
| CMV status adjusted |  |  | ns | F_(1,62)_=2.010 p=0.161 ƞ^2^=0.031 |
| *CD4+EMRA* | 25.13 ± 51.25 | 15.70 ± 29.39 | ns | F_(1,63)_=0.598 p=0.442 ƞ^2^=0.009 |
| Age adjusted |  |  | ns | F_(1,62)_=0.086 p=0.770 ƞ^2^=0.001 |
| VO_2_ max adjusted |  |  | ns | F_(1,62)_=0.024 p=0.878 ƞ^2^=0.000 |
| Body Fat (%) adjusted |  |  | ns | F_(1,62)_=0.445 p=0.507 ƞ^2^=0.007 |
| Lean mass adjusted |  |  | ns | F_(1,62)_=0.201 p=0.655 ƞ^2^=0.003 |
| Fat mass index adjusted |  |  | ns | F_(1,62)_=1.084 p=0.302 ƞ^2^=0.017 |
| CMV status adjusted |  |  | ns | F_(1,62)_=1.249 p=0.268 ƞ^2^=0.020 |
| *CD8+NA* | 141.68 ± 82.63 | **105.59 ± 45.76*** | p<0.05 | **F_(1,63)_=4.690 p=0.034 ƞ^2^=0.069** |
| Age adjusted |  |  | lost | F_(1,62)_=1.543 p=0.219 ƞ^2^=0.024 |
| VO_2_ max adjusted |  |  | lost | F_(1,62)_=1.953 p=0.167 ƞ^2^=0.031 |
| Body Fat (%) adjusted |  |  | lost | F_(1,62)_=3.406 p=0.070 ƞ^2^=0.052 |
| **Lean mass adjusted** |  |  | **maintained** | **F_(1,62)_=4.147 p=0.046 ƞ^2^=0.063** |
| Fat mass index adjusted |  |  | lost | F_(1,62)_=3.793 p=0.056 ƞ^2^=0.058 |
| **CMV status adjusted** |  |  | **maintained** | **F_(1,62)_=4.686 p=0.034 ƞ^2^=0.070** |
| *CD8+CM* | 113.03 ± 74.77 | 105.15 ± 50.82 | ns | F_(1,63)_=0.072 p=0.789 ƞ^2^=0.001 |
| Age adjusted |  |  | ns | F_(1,62)_=0.456 p=0.502 ƞ^2^=0.007 |
| VO_2_ max adjusted |  |  | ns | F_(1,62)_=0.553 p=0.460 ƞ^2^=0.009 |
| Body Fat (%) adjusted |  |  | ns | F_(1,62)_=0.432 p=0.514 ƞ^2^=0.007 |
| Lean mass adjusted |  |  | ns | F_(1,62)_=0.098 p=0.755 ƞ^2^=0.002 |
| Fat mass index adjusted |  |  | ns | F_(1,62)_=0.129 p=0.721 ƞ^2^=0.002 |
| CMV status adjusted |  |  | ns | F_(1,62)_=0.071 p=0.791 ƞ^2^=0.001 |
| *CD8+EM* | 26.42 ± 25.34 | 17.41 ± 16.29 | ns | F_(1,63)_=1.301 p=0.258 ƞ^2^=0.020 |
| Age adjusted |  |  | ns | F_(1,62)_=1.045 p=0.311 ƞ^2^=0.017 |
| VO_2_ max adjusted |  |  | ns | F_(1,62)_=1.284 p=0.262 ƞ^2^=0.020 |
| Body Fat (%) adjusted |  |  | ns | F_(1,62)_=0.993 p=0.323 ƞ^2^=0.016 |
| Lean mass adjusted |  |  | ns | F_(1,62)_=0.720 p=0.399 ƞ^2^=0.011 |
| Fat mass index adjusted |  |  | ns | F_(1,62)_=1.512 p=0.223 ƞ^2^=0.024 |
| CMV status adjusted |  |  | ns | F_(1,62)_=1.311 p=0.257 ƞ^2^=0.021 |
| *CD8+EMRA* | 75.39 ± 88.13 | 69.41 ± 77.96 | ns | F_(1,63)_=0.503 p=0.481 ƞ^2^=0.008 |
| Age adjusted |  |  | ns | F_(1,62)_=1.117 p=0.295 ƞ^2^=0.018 |
| VO_2_ max adjusted |  |  | ns | F_(1,62)_=0.376 p=0.542 ƞ^2^=0.006 |
| Body Fat (%) adjusted |  |  | ns | F_(1,62)_=0.513 p=0.477 ƞ^2^=0.008 |
| Lean mass adjusted |  |  | ns | F_(1,62)_=0.263 p=0.610 ƞ^2^=0.004 |
| Fat mass index adjusted |  |  | ns | F_(1,62)_=0.763 p=0.386 ƞ^2^=0.012 |
| CMV status adjusted |  |  | ns | F_(1,62)_=0.939 p=0.336 ƞ^2^=0.015 |
|  |  |  |  |  |

*Healthy women (n=38) and breast cancer survivors (n=27) were included in the main ANOVA and CMV status adjusted ANCOVA. Data are mean ±* *standard deviation (SD).* ƞ^2^ =partial eta squared. *“*p<0.05” in column 4 = statistically significant between groups in original ANOVA analysis.  *“*ns” = not statistically significant between groups in original ANOVA analysis. “maintained” = statistical significance maintained. “lost” = statistical significance lost. *Statistical significance was considered to be p<0.05. Statistically significant differences between groups have been marked in bold. Abbreviations key: CMV= Cytomegalovirus, VO_2_ max= cardiorespiratory fitness.*

**S. Table 4. T cell activation (proportion of HLA-DR+ cells) after statistical adjustment for age, VO_2_ max, body fat (%), lean mass (kg) fat mass index or CMV status.**

| **Cells and covariates** | **Healthy** *(% of HLA-DR+ cells)* | **Survivors** *(% of HLA-DR+ cells)* | **Original significance** | **Adjusted univariate ANOVA** |
| --- | --- | --- | --- | --- |
| *CD4+ T cells* | 25.28 ± 5.80 | 31.99 ± 15.80 | ns | F_(1,57)_=3.513 p=0.066 ƞ^2^=0.058 |
| Age adjusted |  |  | ns | F_(1,56)_=2.288 p=0.136 ƞ^2^=0.039 |
| VO_2_ max Adjusted |  |  | ns | F_(1,56)_=3.811 p=0.056 ƞ^2^=0.064 |
| Body Fat (%) adjusted |  |  | ns | F_(1,56)_=3.993 p=0.051 ƞ^2^=0.067 |
| Lean mass adjusted |  |  | ns | F_(1,56)_=3.478 p=0.067 ƞ^2^=0.058 |
| Fat mass index adjusted |  |  | ns | F_(1,56)_=1.919 p=0.171 ƞ^2^=0.033 |
| CMV status adjusted |  |  | ns | F_(1,56)_=3.462 p=0.068 ƞ^2^=0.058 |
| *CD4+NA* | 21.57 ± 14.89 | 22.50 ± 14.70 | ns | F_(1,57)_=0.025 p=0.875 ƞ^2^=0.000 |
| Age adjusted |  |  | ns | F_(1,56)_=0.001 p=0.973 ƞ^2^=0.000 |
| VO_2_ max adjusted |  |  | ns | F_(1,56)_=0.048 p=0.828 ƞ^2^=0.001 |
| Body Fat (%) adjusted |  |  | ns | F_(1,56)_=0.037 p=0.848 ƞ^2^=0.001 |
| Lean mass adjusted |  |  | ns | F_(1,56)_=0.011 p=0.916 ƞ^2^=0.000 |
| Fat mass index adjusted |  |  | ns | F_(1,56)_=0.079 p=0.780 ƞ^2^=0.001 |
| CMV status adjusted |  |  | ns | F_(1,56)_=0.025 p=0.876 ƞ^2^=0.000 |
| *CD4+CM* | 27.97 ± 5.97 | **35.22 ± 15.66*** | p<0.05 | **F_(1,57)_=4.160 p=0.046 ƞ^2^=0.068** |
| Age adjusted |  |  | lost | F_(1,56)_=2.291 p=0.136 ƞ^2^=0.039 |
| **VO_2_ max adjusted** |  |  | **maintained** | **F_(1,56)_=4.555 p=0.037 ƞ^2^=0.075** |
| **Body Fat (%) adjusted** |  |  | **maintained** | **F_(1,56)_=4.562 p=0.037 ƞ^2^=0.075** |
| Lean mass adjusted |  |  | lost | F_(1,56)_=3.642 p=0.061 ƞ^2^=0.061 |
| Fat mass index adjusted |  |  | lost | F_(1,56)_=2.848 p=0.097 ƞ^2^=0.048 |
| **CMV status adjusted** |  |  | **maintained** | **F_(1,56)_=4.211 p=0.045 ƞ^2^=0.070** |
| *CD4+EM* | 40.91 ± 9.00 | **54.40 ± 17.29***** | p<0.001 | **F_(1,57)_=12.890 p<0.001 ƞ^2^= 0.184** |
| **Age adjusted** |  |  | **maintained** | **F_(1,56)_=9.295 p=0.004 ƞ^2^=0.142** |
| **VO_2_ max adjusted** |  |  | **maintained** | **F_(1,56)_=12.737 p<0.001 ƞ^2^=0.185** |
| **Body Fat (%) adjusted** |  |  | **maintained** | **F_(1,56)_=12.606 p<0.001 ƞ^2^=0.184** |
| **Lean mass adjusted** |  |  | **maintained** | **F_(1,56)_=11.024 p=0.002 ƞ^2^=0.164** |
| **Fat mass index adjusted** |  |  | **maintained** | **F_(1,56)_=10.342 p=0.002 ƞ^2^=0.156** |
| **CMV status adjusted** |  |  | **maintained** | **F_(1,56)_=12.689 p<0.001 ƞ^2^=0.185** |
| *CD4+EMRA* | 37.43 ± 10.86 | **53.44 ± 20.56***** | p<0.001 | **F_(1,57)_=13.054 p<0.001 ƞ^2^=0.186** |
| **Age adjusted** |  |  | **maintained** | **F_(1,56)_=10.989 p=0.002 ƞ^2^=0.164** |
| **VO_2_ max adjusted** |  |  | **maintained** | **F_(1,56)_=10.363 p=0.002 ƞ^2^=0.156** |
| **Body Fat (%) adjusted** |  |  | **maintained** | **F_(1,56)_=11.940 p=0.001 ƞ^2^=0.176** |
| **Lean mass adjusted** |  |  | **maintained** | **F_(1,56)_=12.544 p<0.001 ƞ^2^=0.183** |
| **Fat mass index adjusted** |  |  | **maintained** | **F_(1,56)_=9.456 p=0.003 ƞ^2^=0.144** |
| **CMV status adjusted** |  |  | **maintained** | **F_(1,56)_=12.895 p<0.001 ƞ^2^=0.187** |
| *CD8+ T cells* | 33.43 ± 9.26 | **43.26 ± 17.93*** | P<0.05 | **F_(1,57)_=6.408 p=0.014 ƞ^2^=0.101** |
| Age adjusted |  |  | lost | F_(1,56)_=2.316 p=0.134 ƞ^2^=0.040 |
| **VO_2_ max adjusted** |  |  | **maintained** | **F_(1,56)_=4.162 p=0.046 ƞ^2^=0.069** |
| **Body Fat (%) adjusted** |  |  | **maintained** | **F_(1,56)_=5.292 p=0.025 ƞ^2^=0.086** |
| **Lean mass adjusted** |  |  | **maintained** | **F_(1,56)_=5.587 p=0.022 ƞ^2^=0.091** |
| Fat mass index adjusted |  |  | lost | F_(1,56)_=3.168 p=0.081 ƞ^2^=0.054 |
| **CMV status adjusted** |  |  | **maintained** | **F_(1,56)_=6.490 p=0.014 ƞ^2^=0.104** |
| *CD8+NA* | 26.49 ± 10.24 | 33.16 ± 15.79 | ns | F_(1,57)_=3.310 p= 0.074 ƞ^2^= 0.055 |
| Age adjusted |  |  | ns | F_(1,56)_=0.468 p=0.497 ƞ^2^=0.008 |
| VO_2_ max adjusted |  |  | ns | F_(1,56)_=2.227 p=0.141 ƞ^2^=0.038 |
| Body Fat (%) adjusted |  |  | ns | F_(1,56)_=2.574 p=0.114 ƞ^2^=0.044 |
| Lean mass adjusted |  |  | ns | F_(1,56)_=2.962 p=0.091 ƞ^2^=0.050 |
| Fat mass index adjusted |  |  | ns | F_(1,56)_=0.954 p=0.333 ƞ^2^=0.017 |
| CMV status adjusted |  |  | ns | F_(1,56)_=3.281 p=0.075 ƞ^2^=0.055 |
| *CD8+CM* | 36.84 ± 10.02 | 44.75 ± 16.73 | ns | F_(1,57)_=3.992 p=0.051 ƞ^2^=0.065 |
| Age adjusted |  |  | ns | F_(1,56)_=1.162 p=0.286 ƞ^2^=0.020 |
| VO_2_ max adjusted |  |  | ns | F_(1,56)_=2.380 p=0.129 ƞ^2^=0.041 |
| Body Fat (%) adjusted |  |  | ns | F_(1,56)_=3.078 p=0.085 ƞ^2^=0.052 |
| Lean mass adjusted |  |  | ns | F_(1,56)_=2.875 p=0.096 ƞ^2^=0.049 |
| Fat mass index adjusted |  |  | ns | F_(1,56)_=1.944 p=0.169 ƞ^2^=0.034 |
| CMV status adjusted |  |  | ns | F_(1,56)_=3.931 p=0.052 ƞ^2^=0.066 |
| *CD8+EM* | 43.89 ± 12.68 | **56.70 ± 17.62**** | P<0.01 | **F_(1,57)_=9.965 p=0.003 ƞ^2^=0.149** |
| **Age adjusted** |  |  | **maintained** | **F_(1,56)_=6.556 p=0.013 ƞ^2^=0.105** |
| **VO_2_ max adjusted** |  |  | **maintained** | **F_(1,56)_=6.708 p=0.012 ƞ^2^=0.107** |
| **Body Fat (%) adjusted** |  |  | **maintained** | **F_(1,56)_=7.601 p=0.008 ƞ^2^=0.120** |
| **Lean mass adjusted** |  |  | **maintained** | **F_(1,56)_=8.183 p=0.006 ƞ^2^=0.127** |
| **Fat mass index adjusted** |  |  | **maintained** | **F_(1,56)_=6.707 p=0.012 ƞ^2^=0.107** |
| **CMV status adjusted** |  |  | **maintained** | **F_(1,56)_=9.947 p=0.003 ƞ^2^=0.151** |
| *CD8+EMRA* | 43.87 ± 11.61 | **54.70 ± 21.04*** | P<0.05 | **F_(1,57)_=4.131 p=0.047 ƞ^2^=0.068** |
| Age adjusted |  |  | lost | F_(1,56)_=2.458 p=0.123 ƞ^2^=0.042 |
| VO_2_ max adjusted |  |  | lost | F_(1,56)_=3.688 p=0.060 ƞ^2^=0.062 |
| **Body Fat (%) adjusted** |  |  | **maintained** | **F_(1,56)_=4.022 p=0.050 ƞ^2^=0.067** |
| Lean mass adjusted |  |  | lost | F_(1,56)_=3.288 p=0.075 ƞ^2^=0.055 |
| Fat mass index adjusted |  |  | lost | F_(1,55)_=2.335 p=0.132 ƞ^2^=0.040 |
| **CMV status adjusted** |  |  | **maintained** | **F_(1,56)_=4.060 p=0.049 ƞ^2^=0.068** |

*Healthy women (n=34) and breast cancer survivors (n=25) were included. Data are mean ±* *standard deviation (SD).* ƞ^2^ =partial eta squared*. “*s” = statistically significant between groups in original ANOVA analysis.  *“*ns” = not statistically significant between groups in original ANOVA analysis. “unchanged” = statistical significance maintained. “lost” = statistical significance lost. *Statistical significance was considered to be p<0.05. Significant differences have been marked in bold. *=p<0.05 **=p<0.01 ***=p<0.001. Abbreviations key: CMV= Cytomegalovirus, VO_2_ max= cardiorespiratory fitness*

**S. Table 5. TSCMs, B cell and NK cell counts adjusted for age, VO_2_ max, body fat (%), lean mass (kg), fat mass index and CMV status.**

| **Cells and covariates** | **Healthy** *(cells/µL)* | **Survivors** *(cells/µL)* | **Original significance** | **Adjusted univariate ANOVA** |
| --- | --- | --- | --- | --- |
| CD4+ TSCMs | 4.00 ± 6.36 | 2.44 ± 1.12 | ns | F_(1,63)_=0.473 p=0.494 ƞ^2^=0.007 |
| Age adjusted |  |  | ns | F_(1,62)_=1.414 p=0.239 ƞ^2^=0.022 |
| VO_2_ max adjusted |  |  | ns | F_(1,62)_=0.001 p=0.981 ƞ^2^=0.000 |
| Body Fat (%) adjusted |  |  | ns | F_(1,62)_=0.060 p=0.807 ƞ^2^=0.001 |
| Lean mass adjusted |  |  | ns | F_(1,62)_=0.967 p=0.329 ƞ^2^=0.015 |
| Fat mass index adjusted |  |  | ns | F_(1,62)_=0.095 p=0.759 ƞ^2^=0.002 |
| CMV status adjusted |  |  | ns | F_(1,62)_=0.465 p=0.498 ƞ^2^=0.007 |
| CD8+ TSCMs | 0.50 ± 0.65 | 0.22 ± 0.42 | ns | F_(1,63)_=3.531 p=0.065 ƞ^2^=0.053 |
| Age adjusted |  |  | ns | F_(1,62)_=2.563 p=0.114 ƞ^2^=0.040 |
| VO_2_ max adjusted |  |  | ns | F_(1,62)_=1.866 p=0.177 ƞ^2^=0.029 |
| Body Fat (%) adjusted |  |  | ns | F_(1,62)_=2.259 p=0.138 ƞ^2^=0.035 |
| Lean mass adjusted |  |  | ns | F_(1,62)_=3.297 p=0.074 ƞ^2^=0.050 |
| Fat mass index adjusted |  |  | ns | F_(1,62)_=2.711 p=0.105 ƞ^2^=0.042 |
| CMV status adjusted |  |  | ns | F_(1,62)_=3.687 p=0.059 ƞ^2^=0.056 |
| CD19+ B cells | 27.29 ± 23.89 | 39.96 ± 57.74 | ns | F_(1,59)_=0.133 p=0.717 ƞ^2^= 0.002 |
| Age Adjusted |  |  | ns | F_(1,58)_=0.001 p=0.980 ƞ^2^=0.000 |
| VO2 max Adjusted |  |  | ns | F_(1,58)_=0.517 p=0.475 ƞ^2^=0.009 |
| Body Fat (%) adjusted |  |  | ns | F_(1,58)_=0.427 p=0.516 ƞ^2^=0.007 |
| Lean mass adjusted |  |  | ns | F_(1,58)_=0.179 p=0.674 ƞ^2^=0.003 |
| Fat mass index adjusted |  |  | ns | F_(1,58)_=0.002 p=0.964 ƞ^2^=0.000 |
| CMV status adjusted |  |  | ns | F_(1,58)_=0.129 p=0.721 ƞ^2^=0.002 |
| Plasmablasts | 1.17 ± 1.15 | 1.27 ± 1.28 | ns | F_(1,59)_=0.021 p=0.885 ƞ^2^=0.000 |
| Age Adjusted |  |  | ns | F_(1,58)_=0.521 p=0.473 ƞ^2^=0.009 |
| VO2 max Adjusted |  |  | ns | F_(1,58)_=0.361 p=0.550 ƞ^2^=0.006 |
| Body Fat (%) adjusted |  |  | ns | F_(1,58)_=0.169 p=0.683 ƞ^2^=0.003 |
| Lean mass adjusted |  |  | ns | F_(1,58)_=0.003 p=0.957 ƞ^2^=0.000 |
| Fat mass index adjusted |  |  | ns | F_(1,58)_=0.000 p=0.999 ƞ^2^=0.000 |
| CMV status adjusted |  |  | ns | F_(1,58)_=0.024 p=0.878 ƞ^2^=0.000 |
| Memory B cells | 3.91 ± 3.97 | 5.35 ± 8.60 | ns | F_(1,59)_=0.006 p=0.936 ƞ^2^=0.000 |
| Age Adjusted |  |  | ns | F_(1,58)_=0.008 p=0.931 ƞ^2^=0.000 |
| VO2 max Adjusted |  |  | ns | F_(1,58)_=0.222 p=0.639 ƞ^2^=0.004 |
| Body Fat (%) adjusted |  |  | ns | F_(1,58)_=0.143 p=0.706 ƞ^2^=0.002 |
| Lean mass adjusted |  |  | ns | F_(1,58)_=0.002 p=0.962 ƞ^2^=0.000 |
| Fat mass index adjusted |  |  | ns | F_(1,58)_=0.002 p=0.969 ƞ^2^=0.000 |
| CMV status adjusted |  |  | ns | F_(1,58)_=0.006 p=0.940 ƞ^2^=0.000 |
| Immature B cells | 0.71 ± 0.99 | **2.35 ± 4.79*** | P<0.05 | **F_(1,59)_=4.417 p=0.040 ƞ^2^=0.070** |
| Age Adjusted |  |  | lost | F_(1,58)_=3.148 p=0.081 ƞ^2^=0.051 |
| **VO2 max Adjusted** |  |  | **maintained** | **F_(1,58)_=4.586 p=0.036 ƞ^2^=0.073** |
| **Body Fat (%) adjusted** |  |  | **maintained** | **F_(1,58)_=4.627 p=0.036 ƞ^2^=0.074** |
| **Lean mass adjusted** |  |  | **maintained** | **F_(1,58)_=5.303 p=0.025 ƞ^2^=0.084** |
| Fat mass index adjusted |  |  | lost | F_(1,58)_=2.585 p=0.113 ƞ^2^=0.043 |
| **CMV status adjusted** |  |  | **maintained** | **F_(1,58)_=4.457 p=0.039 ƞ^2^=0.071** |
| Naive B cells | 21.54 ± 19.67 | 30.81 ± 49.12 | ns | F_(1,59)_=0.008 p=0.929 ƞ^2^=0.000 |
| Age Adjusted |  |  | ns | F_(1,58)_=0.116 p=0.735 ƞ^2^=0.002 |
| VO2 max Adjusted |  |  | ns | F_(1,58)_=0.159 p=0.691 ƞ^2^=0.003 |
| Body Fat (%) adjusted |  |  | ns | F_(1,58)_=0.112 p=0.739 ƞ^2^=0.002 |
| Lean mass adjusted |  |  | ns | F_(1,58)_=0.034 p=0.855 ƞ^2^=0.001 |
| Fat mass index adjusted |  |  | ns | F_(1,58)_=0.065 p=0.800 ƞ^2^=0.001 |
| CMV status adjusted |  |  | ns | F_(1,58)_=0.006 p=0.936 ƞ^2^=0.000 |
| NK cells | 61.43 ± 55.28 | **127.31 ± 106.19**** | P<0.01 | **F_(1,59)_=11.403 p=0.001 ƞ^2^=0.162** |
| **Age adjusted** |  |  | **maintained** | **F_(1,58)_=8.272 p=0.006 ƞ^2^=0.125** |
| **VO_2_ max adjusted** |  |  | **maintained** | **F_(1,58)_=17.547 p<0.001 ƞ^2^=0.232** |
| **Body Fat (%) adjusted** |  |  | **maintained** | **F_(1,58)_=15.001 p<0.001 ƞ^2^=0.205** |
| **Lean mass adjusted** |  |  | **maintained** | **F_(1,58)_=8.753 p=0.004 ƞ^2^=0.131** |
| **Fat mass index adjusted** |  |  | **maintained** | **F_(1,58)_=12.246 p<0.001 ƞ^2^=0.174** |
| **CMV status adjusted** |  |  | **maintained** | **F_(1,58)_=11.194 p=0.001 ƞ^2^=0.162** |
| CD16+ Effector NK cells | 43.66 ± 52.59 | **106.54 ± 98.05***** | P<0.001 | **F_(1,59)_=13.600 p=0.000 ƞ^2^=0.187** |
| **Age adjusted** |  |  | **maintained** | **F_(1,58)_=11.340 p=0.001 ƞ^2^=0.164** |
| **VO_2_ max adjusted** |  |  | **maintained** | **F_(1,58)_=21.386 p<0.001 ƞ^2^=0.269** |
| **Body Fat (%) adjusted** |  |  | **maintained** | **F_(1,58)_=18.214 p<0.001 ƞ^2^=0.239** |
| **Lean mass adjusted** |  |  | **maintained** | **F_(1,58)_=10.726 p=0.002 ƞ^2^=0.156** |
| **Fat mass index adjusted** |  |  | **maintained** | **F_(1,58)_=15.373 p<0.001 ƞ^2^=0.210** |
| **CMV status adjusted** |  |  | **maintained** | **F_(1,58)_=13.383 p<0.001 ƞ^2^=0.187** |
| CD16- Regulatory NK cells | 17.71 ± 13.60 | 20.73 ± 13.35 | ns | F_(1,59)_=1.840 p=0.180 ƞ^2^=0.030 |
| Age adjusted |  |  | ns | F_(1,58)_=0.606 p=0.439 ƞ^2^=0.010 |
| VO_2_ max adjusted |  |  | ns | F_(1,58)_=2.089 p=0.154 ƞ^2^=0.035 |
| Body Fat (%) adjusted |  |  | ns | F_(1,58)_=2.115 p=0.151 ƞ^2^=0.035 |
| Lean mass adjusted |  |  | ns | F_(1,58)_=1.184 p=0.281 ƞ^2^=0.020 |
| Fat mass index adjusted |  |  | ns | F_(1,58)_=1.290 p=0.261 ƞ^2^=0.022 |
| CMV status adjusted |  |  | ns | F_(1,58)_=1.774 p=0.188 ƞ^2^=0.030 |

*Healthy women (n=38 for TSCMs; n=35 for B cell and NK cell subsets) and breast cancer survivors (n=27 for TSCMs; n=26 for B cell and NK cell subsets) were Data are mean ±* *standard deviation (SD).* ƞ^2^ =partial eta squared*. “*s” = statistically significant between groups in original ANOVA analysis.  *“*ns” = not statistically significant between groups in original ANOVA analysis. “unchanged” = statistical significance maintained. “lost” = statistical significance lost. *Statistical significance was considered to be p<0.05. Significant differences have been marked in bold. *=p<0.05 **=p<0.01 ***=p<0.001. Abbreviations key: CMV= Cytomegalovirus, VO_2_ max= cardiorespiratory fitness.*

**S. Table 6. Linear regression analyses between activation (proportion of HLA-DR+ cells) of CD4+ and CD8+ effector memory T cell subsets and Fat Mass Index, after statistical adjustment for age, VO_2_ max, lean mass (kg) or CMV status.**

| **Variables controlled for after original correlation** | **Original correlation**  **significance** | **Adjusted linear regression** |
| --- | --- | --- |
| *CD4+EM vs Fat Mass Index* | r=0.233, p=0.076 | F_(1,57)_ = 3.275 p=0.076 R=0.233 R^2^=0.054 R^2^_Adjusted_=0.038 |
| Age adjusted | maintained | F_(1,56)_ = 1.717 p=0.195 R=0.289 R^2^=0.084 R^2^_Adjusted_=0.051 R^2^_Change_=0.028 |
| VO_2_ max adjusted | maintained | F_(1,56)_ = 2.519 p=0.118 R=0.237 R^2^=0.056 R^2^_Adjusted_=0.022 R^2^_Change_=0.042 |
| **Lean mass adjusted** | **gained** | **F_(1,56)_ = 4.594 p=0.036 R=0.316 R^2^=0.100 R^2^_Adjusted_=0.067 R^2^_Change_=0.074** |
| CMV status adjusted | maintained | F_(1,56)_ = 3.311 p=0.074 R=0.236 R^2^=0.056 R^2^_Adjusted_=0.022 R^2^_Change_=0.056 |
| *CD4+EMRA vs Fat Mass Index* | **r=0.305, p=0.019** | **F_(1,57)_ = 5.830 p=0.019 R=0.305 R^2^=0.093 R^2^_Adjusted_=0.077** |
| **Age adjusted** | **maintained** | **F_(1,56)_ = 4.114 p=0.047 R=0.322 R^2^=0.104 R^2^_Adjusted_=0.072 R^2^_Change_=0.066** |
| VO_2_ max adjusted | lost | F_(1,56)_ = 3.957 p=0.052 R=0.306 R^2^=0.094 R^2^_Adjusted_=0.061 R^2^_Change_=0.064 |
| **Lean mass adjusted** | **maintained** | **F_(1,56)_ = 6.685 p=0.012 R=0.333 R^2^=0.111 R^2^_Adjusted_=0.079 R^2^_Change_=0.106** |
| **CMV status adjusted** | **maintained** | **F_(1,56)_ = 5.576 p=0.022 R=0.305 R^2^=0.093 R^2^_Adjusted_=0.060 R^2^_Change_=0.090** |
| *CD8+EM vs Fat Mass Index* | **r=0.309, p=0.017** | **F_(1,57)_ = 6.014 p=0.017 R=0.309 R^2^=0.095 R^2^_Adjusted_=0.080** |
| **Age adjusted** | **maintained** | **F_(1,56)_ = 4.222 p=0.045 R=0.327 R^2^=0.107 R^2^_Adjusted_=0.075 R^2^_Change_=0.067** |
| VO_2_ max adjusted | lost | F_(1,56)_ = 3.128 p=0.082 R=0.310 R^2^=0.096 R^2^_Adjusted_=0.064 R^2^_Change_=0.050 |
| **Lean mass adjusted** | **maintained** | **F_(1,56)_ = 8.705 p=0.005 R=0.414 R^2^=0.171 R^2^_Adjusted_=0.142 R^2^_Change_=0.129** |
| **CMV status adjusted** | **maintained** | **F_(1,56)_ = 5.459 p=0.023 R=0.316 R^2^=0.100 R^2^_Adjusted_=0.068 R^2^_Change_=0.088** |
| *CD8+EMRA vs Fat Mass Index* | **r=0.320, p=0.014** | **F_(1,57)_ = 6.480 p=0.014 R=0.320 R^2^=0.102 R^2^_Adjusted_=0.086** |
| Age adjusted | **maintained** | **F_(1,56)_ = 4.622 p=0.036 R=0.336 R^2^=0.113 R^2^_Adjusted_=0.081 R^2^_Change_=0.073** |
| VO_2_ max adjusted | **maintained** | **F_(1,56)_ = 5.930 p=0.018 R=0.333 R^2^=0.111 R^2^_Adjusted_=0.079 R^2^_Change_=0.094** |
| Lean mass adjusted | **maintained** | **F_(1,56)_ = 8.176 p=0.006 R=0.377 R^2^=0.142 R^2^_Adjusted_=0.112 R^2^_Change_=0.125** |
| CMV status adjusted | **maintained** | **F_(1,56)_ = 6.208 p=0.016 R=0.320 R^2^=0.102 R^2^_Adjusted_=0.070 R^2^_Change_=0.100** |

*All healthy women and breast cancer survivors (n= 34 + 25= 59) were included in the hierarchical multiple linear regression analyses.*  “maintained” = statistical significance maintained. “lost” = statistical significance lost. “gained” = statistical significance gained. *Statistical significance was considered to be p<0.05. Significant differences have been marked in bold. Abbreviations key: CMV= Cytomegalovirus, VO_2_ max= cardiorespiratory fitness.*

**S. Table 7. Comparison between original analyses (n=38 heathy women) and alternative analyses where healthy women under the age of 35 years (n=10) were excluded to align the age-range of both groups.**

Eligibility criteria differed between the study groups, such as the age range (healthy women: 25-69 years vs. survivors: 35-69 years). Including healthy participants below 35 years old (n=10) is an important part of this study due to the need for (a) dataset heterogeneity to explore relationships between participant characteristics and immunological variables and (b) to maintain statistical power for ANCOVA and linear regression analysis. However, a comparison has been presented below between the original analyses (n=38 healthy women vs. n=27 survivors) and alternative analyses when excluding n=10 healthy women <35 years (n=28 healthy women vs. n=27 survivors). See legend below table for colour coding key (i.e., green, blue, red).

| **Characteristics** | **Healthy** (original analysis) | **Healthy**  (removing <35 years) | **Survivors** | **Univariate ANOVA**  (original analysis) | **Univariate ANOVA**  (removing <35 years) | **Change in significance between 1 & 2** | |
| --- | --- | --- | --- | --- | --- | --- | --- |
| Sample size | 38 | 28 | 27 |  |  |  | |
| Age (years) | 45 ± 11 | 49 ± 8 | 56 ± 6 | F_(1,63)_=25.914 p<0.001 ƞ^2^=0.291 | F_(1,53)_=14.591 p<0.001 ƞ^2^=0.216 | | No |
| Height (m) | 1.67 ± 0.75 | 1.67 ± 0.08 | 1.63 ± 0.06 | F_(1,63)_=7.042 p=0.010 ƞ^2^=0.101 | F_(1,53)_=5.512 p=0.023 ƞ^2^=0.094 | | No |
| Body mass (kg) | 70.4 ± 17.2 | 69.2 ± 14.8 | 68.5 ± 9.9 | F_(1,63)_=0.081 p=0.777 ƞ^2^=0.001 | F_(1,53)_=0.000 p=0.995 ƞ^2^=0.000 | | No |
| BMI (kg/m^2^) | 25.1 ± 5.0 | 24.7 ± 4.3 | 25.9 ± 3.4 | F_(1,63)_=1.057 p=0.308 ƞ^2^=0.017 | F_(1,53)_=1.730 p=0.194 ƞ^2^=0.032 | | No |
| VO_2_ max (mL•kg^-1^•min^-1^) | 36.2 ± 8.5 | 34.3 ± 6.2 | 28.8 ± 5.0 | F_(1,63)_=17.858 p<0.001 ƞ^2^=0.221 | F_(1,53)_=12.105 p=0.001 ƞ^2^=0.186 | | No |
| Body fat (%) | 32.7 ± 6.4 | 33.7 ± 4.9 | 36.3 ± 5.3 | F_(1,63)_=5.462 p=0.023 ƞ^2^=0.080 | F_(1,53)_=2.999 p=0.089 ƞ^2^=0.054 | | Yes |
| Lean mass (kg) | 48.4 ± 15.8 | 45.7 ± 8.1 | 42.3 ± 5.0 | F_(1,63)_=4.753 p=0.033 ƞ^2^=0.070 | F_(1,53)_=3.060 p=0.086 ƞ^2^=0.055 | | Yes |
| Fat mass (kg) | 22.6 ± 7.9 | 23.5 ± 8.1 | 25.2 ± 5.9 | F_(1,63)_=3.203 p=0.078 ƞ^2^=0.048 | F_(1,53)_=1.308 p=0.258 ƞ^2^=0.024 | | No |
| Fat mass index (kg/m^2^) | 8.1 ± 2.7 | 8.4 ± 2.8 | 9.5 ± 2.2 | F_(1,63)_=6.801 p=0.011 ƞ^2^=0.097 | F_(1,53)_=3.648 p=0.062 ƞ^2^=0.064 | | Yes |
| Sitting time (min/week) | 2,502 ± 916 | 2,496 ± 910 | 1,883 ± 958 | F_(1,61)_=6.779 p=0.012 ƞ^2^=0.100 | F_(1,52)_=5.811 p=0.019 ƞ^2^=0.101 | | No |
| Light activity time (min/w) | 588 ± 834 | 667 ± 948 | 677 ± 724 | F_(1,61)_=0.174 p=0.678 ƞ^2^=0.003 | F_(1,52)_=0.086 p=0.770 ƞ^2^=0.002 | | No |
| Moderate activity time (min/w) | 399 ± 659 | 477 ± 744 | 481 ± 607 | F_(1,61)_=0.057 p=0.812 ƞ^2^=0.001 | F_(1,52)_=0.003 p=0.960 ƞ^2^=0.000 | | No |
| Vigorous activity time (min/w) | 302 ± 572 | 371 ± 646 | 103 ± 198 | F_(1,61)_=13.095 p<0.001 ƞ^2^=0.177 | F_(1,52)_=18.516 p<0.001 ƞ^2^=0.263 | | No |
| CMV+ individuals | 17 (44.7%) | 12 (42.9%) | 12 (44.4%) | F_(1,63)_=0.001 p=0.982 ƞ^2^<0.001 | F_(1,53)_=0.014 p=0.908 ƞ^2^=0.000 | | No |
| CMV IgG (IU/ml)^1^ | 14.79 ± 6.44 | 16.11 ± 7.04 | 17.28 ± 5.37 | F_(1,27)_=1.296 p=0.265 ƞ^2^=0.046 | F_(1,22)_=0.290 p=0.596 ƞ^2^=0.013 | | No |

**Leukocyte differential** (cells x10^9^/L)

| Total leukocytes | 5.27 ± 1.52 | 5.28 ± 1.57 | 5.06 ± 1.33 | F_(1,63)_=0.253 p=0.617 ƞ^2^=0.004 | F_(1,53)_=0.247 p=0.621 ƞ^2^=0.005 | No |
| --- | --- | --- | --- | --- | --- | --- |
| Lymphocytes | 1.55 ± 0.48 | 1.57 ± 0.53 | 1.46 ± 0.38 | F_(1,63)_=0.630 p=0.430 ƞ^2^=0.010 | F_(1,53)_=0.726 p=0.398 ƞ^2^=0.014 | No |
| Monocytes^2^ | 0.41 ± 0.20 | 0.44 ± 0.21 | 0.41 ± 0.18 | F_(1,63)_=0.008 p=0.927 ƞ^2^=0.000 | F_(1,53)_=0.204 p=0.654 ƞ^2^=0.004 | No |
| Neutrophils | 3.31 ± 1.19 | 3.28 ± 1.18 | 3.17 ± 1.13 | F_(1,63)_=0.202 p=0.655 ƞ^2^=0.003 | F_(1,53)_=0.122 p=0.728 ƞ^2^=0.002 | No |

**T cell analysis** (cells/$\mu$L or % cells)

| CD4+ total (T cells) | 844.08 ± 282.90 | 839.04 ± 286.39 | 873.96 ± 260.47 | F_(1,63)_=0.340 p=0.562 ƞ^2^=0.005 | F_(1,53)_=0.405 p=0.527 ƞ^2^=0.008 | No |
| --- | --- | --- | --- | --- | --- | --- |
| CD4+NA | 408.11 ± 236.63 | 412.50 ± 235.33 | 371.85 ± 208.50 | F_(1,63)_=0.878 p=0.352 ƞ^2^=0.014 | F_(1,53)_=0.855 p=0.359 ƞ^2^=0.016 | No |
| CD4+CM | 345.55 ± 160.75 | 334.57 ± 173.25 | 417.11 ± 109.48 | F_(1,63)_=5.039 p=0.028 ƞ^2^=0.074 | F_(1,53)_=5.978 p=0.018 ƞ^2^=0.10 | No |
| CD4+EM | 65.29 ± 58.66 | 64.46 ± 62.00 | 69.26 ± 38.39 | F_(1,63)_=1.600 p=0.211 ƞ^2^=0.025 | F_(1,53)_=1.943 p=0.169 ƞ^2^=0.035 | No |
| CD4+EMRA | 25.13 ± 51.25 | 27.61 ± 57.42 | 15.70 ± 29.39 | F_(1,63)_=0.598 p=0.442 ƞ^2^=0.009 | F_(1,53)_=0.684 p=0.412 ƞ^2^=0.013 | No |
| CD8+total (T cells) | 356.50 ± 178.93 | 366.61 ± 200.52 | 297.44 ± 129.20 | F_(1,63)_=2.018 p=0.160 ƞ^2^=0.031 | F_(1,53)_=1.624 p=0.208 ƞ^2^=0.030 | No |
| CD8+NA | 141.68 ± 82.63 | 141.11 ± 94.81 | 105.59 ± 45.76 | F_(1,63)_=4.690 p=0.034 ƞ^2^=0.069 | F_(1,53)_=2.624 p=0.111 ƞ^2^=0.047 | Yes |
| CD8+CM | 113.03 ± 74.77 | 108.71 ± 79.76 | 105.15 ± 50.82 | F_(1,63)_=0.072 p=0.789 ƞ^2^=0.001 | F_(1,53)_=0.482 p=0.491 ƞ^2^=0.009 | No |
| CD8+EM | 26.42 ± 25.34 | 28.25 ± 27.87 | 17.41 ± 16.29 | F_(1,63)_=1.301 p=0.258 ƞ^2^=0.020 | F_(1,53)_=1.035 p=0.314 ƞ^2^=0.019 | No |
| CD8+EMRA | 75.39 ± 88.13 | 88.50 ± 98.02 | 69.41 ± 77.96 | F_(1,63)_=0.503 p=0.481 ƞ^2^=0.008 | F_(1,53)_=1.320 p=0.256 ƞ^2^=0.024 | No |
| Activ CD4+ total (%) | 25.28 ± 5.80 | 25.02 ± 6.05 | 31.99 ± 15.80 | F_(1,57)_=3.513 p=0.066 ƞ^2^=0.058 | F_(1,49)_=3.245 p=0.078 ƞ^2^=0.062 | No |
| Activ CD4+NA (%) | 21.57 ± 14.89 | 18.80 ± 5.84 | 22.50 ± 14.70 | F_(1,57)_=0.025 p=0.875 ƞ^2^=0.000 | F_(1,49)_=0.183 p=0.671 ƞ^2^=0.004 | No |
| Activ CD4+CM (%) | 27.97 ± 5.97 | 27.98 ± 6.27 | 35.22 ± 15.66 | F_(1,57)_=4.160 p=0.046 ƞ^2^=0.068 | F_(1,49)_=3.390 p=0.072 ƞ^2^=0.065 | Yes |
| Activ CD4+EM (%) | 40.91 ± 9.00 | 41.12 ± 9.35 | 54.40 ± 17.29 | F_(1,57)_=12.890 p<0.001 ƞ^2^=0.184 | F_(1,49)_=10.334 p=0.002 ƞ^2^=0.174 | No |
| Activ CD4+EMRA (%) | 37.43 ± 10.86 | 36.94 ± 11.53 | 53.44 ± 20.56 | F_(1,57)_=13.054 p<0.001 ƞ^2^=0.186 | F_(1,49)_=11.838 p=0.001 ƞ^2^=0.195 | No |
| Activ CD8+total (%) | 33.43 ± 9.26 | 35.46 ± 9.50 | 43.26 ± 17.93 | F_(1,57)_=6.408 p=0.014 ƞ^2^=0.101 | F_(1,49)_=2.773 p=0.102 ƞ^2^=0.054 | Yes |
| Activ CD8+NA (%) | 26.49 ± 10.24 | 28.34 ± 11.04 | 33.16 ± 15.79 | F_(1,57)_=3.310 p=0.074 ƞ^2^=0.055 | F_(1,49)_=1.160 p=0.287 ƞ^2^=0.023 | No |
| Activ CD8+CM (%) | 36.84 ± 10.02 | 38.96 ± 9.56 | 44.75 ± 16.73 | F_(1,57)_=3.992 p=0.051 ƞ^2^=0.065 | F_(1,49)_=1.395 p=0.243 ƞ^2^=0.028 | No |
| Activ CD8+EM (%) | 43.89 ± 12.68 | 45.45 ± 12.03 | 56.70 ± 17.62 | F_(1,57)_=9.965 p=0.003 ƞ^2^=0.149 | F_(1,49)_=6.622 p=0.013 ƞ^2^=0.119* | No |
| Activ CD8+EMRA (%) | 43.87 ± 11.61 | 45.03 ± 12.28 | 54.70 ± 21.04 | F_(1,57)_=4.131 p=0.047 ƞ^2^=0.068 | F_(1,49)_=2.539 p=0.118 ƞ^2^=0.049 | Yes |

**Other cells** (cells/$\mu$L)

| CD4/CD8 ratio | 2.82 ± 1.47 | 2.83 ± 1.58 | 3.73 ± 2.66 | F_(1,63)_=2.945 p=0.091 ƞ^2^= 0.045 | F_(1,53)_=2.480 p=0.121 ƞ^2^= 0.045 | No |
| --- | --- | --- | --- | --- | --- | --- |
| CD4+ TSCMs | 4.00 ± 6.36 | 4.50 ± 7.33 | 2.44 ± 1.12 | F_(1,63)_=0.473 p=0.494 ƞ^2^= 0.007 | F_(1,53)_=0.594 p=0.444 ƞ^2^= 0.011 | No |
| CD8+ TSCMs | 0.50 ± 0.65 | 0.46 ± 0.69 | 0.22 ± 0.42 | F_(1,63)_=3.531 p=0.065 ƞ^2^= 0.053 | F_(1,53)_=1.994 p=0.164 ƞ^2^= 0.036 | No |
| CD19+ total (B cells) | 27.29 ± 23.89 | 28.70 ± 26.82 | 39.96 ± 57.74 | F_(1,59)_=0.133 p=0.717 ƞ^2^= 0.002 | F_(1,51)_=0.064 p=0.802 ƞ^2^= 0.001 | No |
| Plasmablasts | 1.17 ± 1.15 | 1.11 ± 1.16 | 1.27 ± 1.28 | F_(1,59)_=0.021 p=0.885 ƞ^2^= 0.000 | F_(1,51)_=0.137 p=0.713 ƞ^2^= 0.003 | No |
| Memory B cells | 3.91 ± 3.97 | 3.85 ± 4.22 | 5.35 ± 8.60 | F_(1,59)_=0.006 p=0.936 ƞ^2^= 0.000 | F_(1,51)_=0.005 p=0.942 ƞ^2^= 0.000 | No |
| Immature B cells | 0.71 ± 0.99 | 0.74 ± 1.10 | 2.35 ± 4.79 | F_(1,59)_=4.417 p=0.040 ƞ^2^= 0.070 | F_(1,51)_=3.599 p=0.063 ƞ^2^= 0.066 | Yes |
| Naive B cells | 21.54 ± 19.67 | 23.07 ± 22.00 | 30.81 ± 49.11 | F_(1,59)_=0.008 p=0.929 ƞ^2^= 0.000 | F_(1,51)_=0.006 p=0.940 ƞ^2^= 0.000 | No |
| CD56+ total (NK cells) | 61.43 ± 55.28 | 55.67 ± 50.11 | 127.31 ± 106.19 | F_(1,59)_=11.403 p=0.001 ƞ^2^= 0.162 | F_(1,51)_=12.472 p<0.001 ƞ^2^= 0.243 | No |
| CD16+ Effector cells | 43.66 ± 52.59 | 36.44 ± 47.74 | 106.54 ± 98.05 | F_(1,59)_=13.600 p<0.001 ƞ^2^= 0.187 | F_(1,51)_=16.363 p<0.001 ƞ^2^= 0.243 | No |
| CD16- Regulatory cells | 17.71 ± 13.60 | 19.15 ± 14.55 | 20.73 ± 13.35 | F_(1,59)_=1.840 p=0.180 ƞ^2^= 0.030 | F_(1,51)_=0.830 p=0.367 ƞ^2^= 0.016 | No |

*Data are mean ± standard deviation (SD) for (1) original analyses: n=38 healthy women vs. n=27 survivors for most of the variables and (2) alternative analyses were <35-year-old healthy women have been excluded: n=28 healthy women vs. n=27 survivors for most of the variables. Univariate ANOVA was performed using log10 transformed data. Statistical significance was considered as p<0.05. Abbreviations: Activ= Activation (HLA-DR+), BMI= body mass index, CMV= Cytomegalovirus, IgG= Immunoglobulin G, VO_2_max= cardiorespiratory fitness. Notes: ^1^CMV IgG for CMV seropositive individuals. ^2^Monocytes refer to the “Mixed cells” fraction from an automated haematology analyser: <10% correspond to basophils and eosinophils. Colour key: Green text shows differences that were statistically significant in our original analysis that have remained statistically significant with the alternative analysis. Blue text shows differences that were statistically significant in our original analysis but are now close to statistical significance (p>0.05 but p<0.1) with the alternative analysis. Red text shows differences that were statistically significant in our original analysis but are now lost (p>0.1) in the alternative analysis.*

In summary, five of the participant characteristics that showed a statistically significant difference between groups in the original analyses remain statistically significant when n=10 healthy women are excluded (age, height, VO_2_ max, sitting time and vigorous activity time). However, group differences among three other variables lose statistical significance (body fat, lean mass, fat mass index) most likely due to lower statistical power with the smaller sample size. For immunological variables, in summary, statistical significance was maintained when removing n=10 healthy women under the age of 35 years for all comparisons of leukocyte differential between groups, most comparisons of T cells between groups, and most comparisons between groups for TSCMs, B cells and NK cells. Overall, the main findings are maintained with both approaches, as, for example, even with the smaller sample size, we still detect among survivors, significantly higher levels of central memory CD4+ T cells and higher activation levels of effector memory CD4+ and CD8+ T cell subsets, as well as higher levels of total and effector NK cells.

**Supplementary Figures**

***S. Figure 1. T cell flow cytometry analysis****.*

*
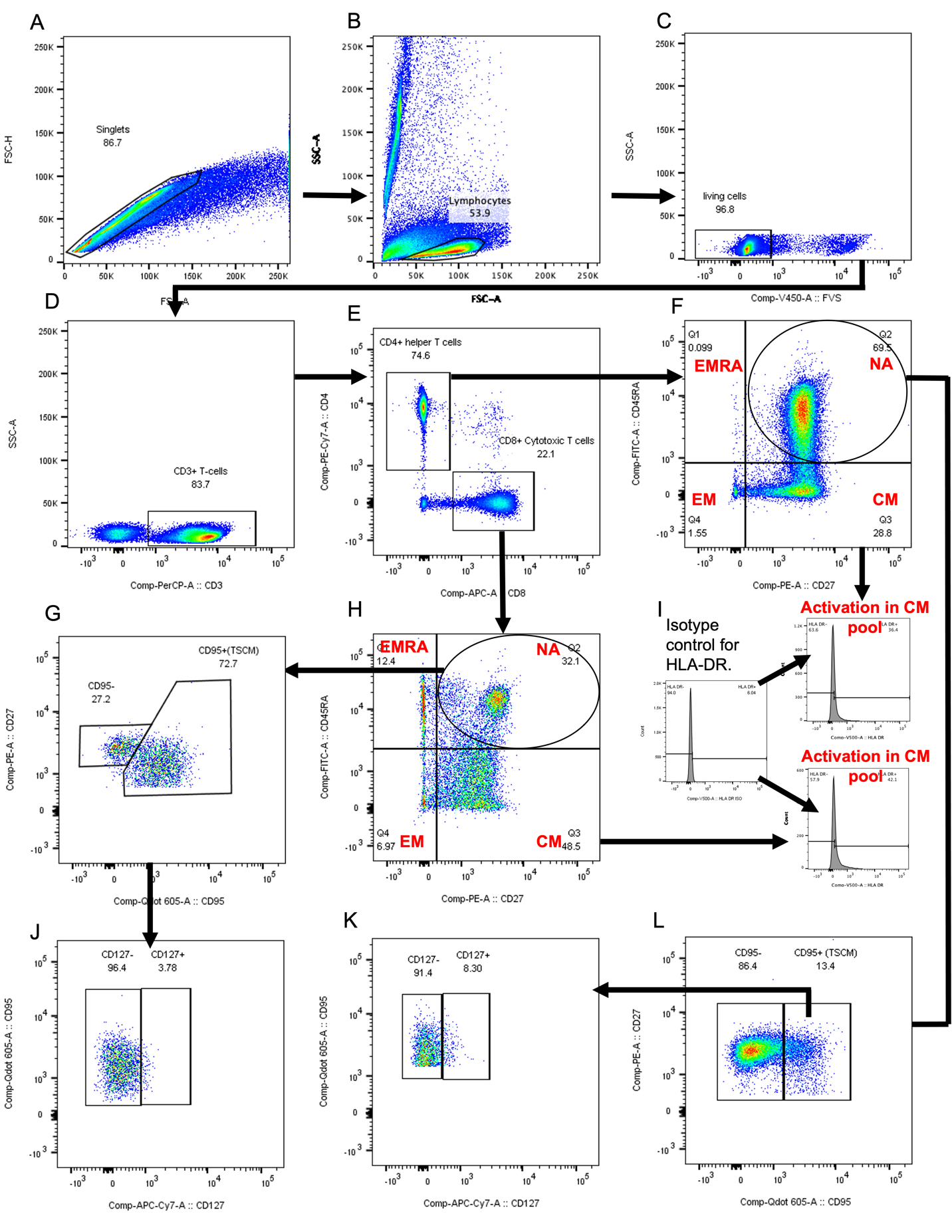
*

*Representative flow cytometry analysis with a sample from a healthy participant. Singlets were gated to discard doublets* ***(A)****. FSC versus SSC was plotted to identify lymphocytes* ***(B****). Viable cells were identified* ***(C)****. CD3+ T cells were identified* ***(D)****. CD4+ helper and CD8+ cytotoxic cells were identified* ***(E).*** *Subsets were gated using CD27 and CD45RA to discriminate by maturation/differentiation (****F*** *for CD4+ and* ***H*** *for CD8+) into Naïve (NA), Central Memory (CM), Effector Memory (EM) and Effector Memory expressing CD45RA (EMRA). All subsets were assessed for HLA-DR expression, as a marker of activation. Using an isotype control tube ≈ 5% HLA-DR+ cells were determined, and gate positioning was used on other subsets to determine activation (expressed as Median Fluorescence Intensity (MFI))* ***(I)****. CD4+ and CD8+ stem cell like T cells (TSCMs) were identified by CD95 expression from the naïve T cell pools (****G*** *for CD8+ and* ***L*** *for CD4+), and CD95+ cells were gated against CD127, as both have been identified as markers of TSCMs (****J*** *for CD8+ and* ***K*** *for CD4+) .*

**S. Figure 2. B cells and NK cells flow cytometry analysis**

*
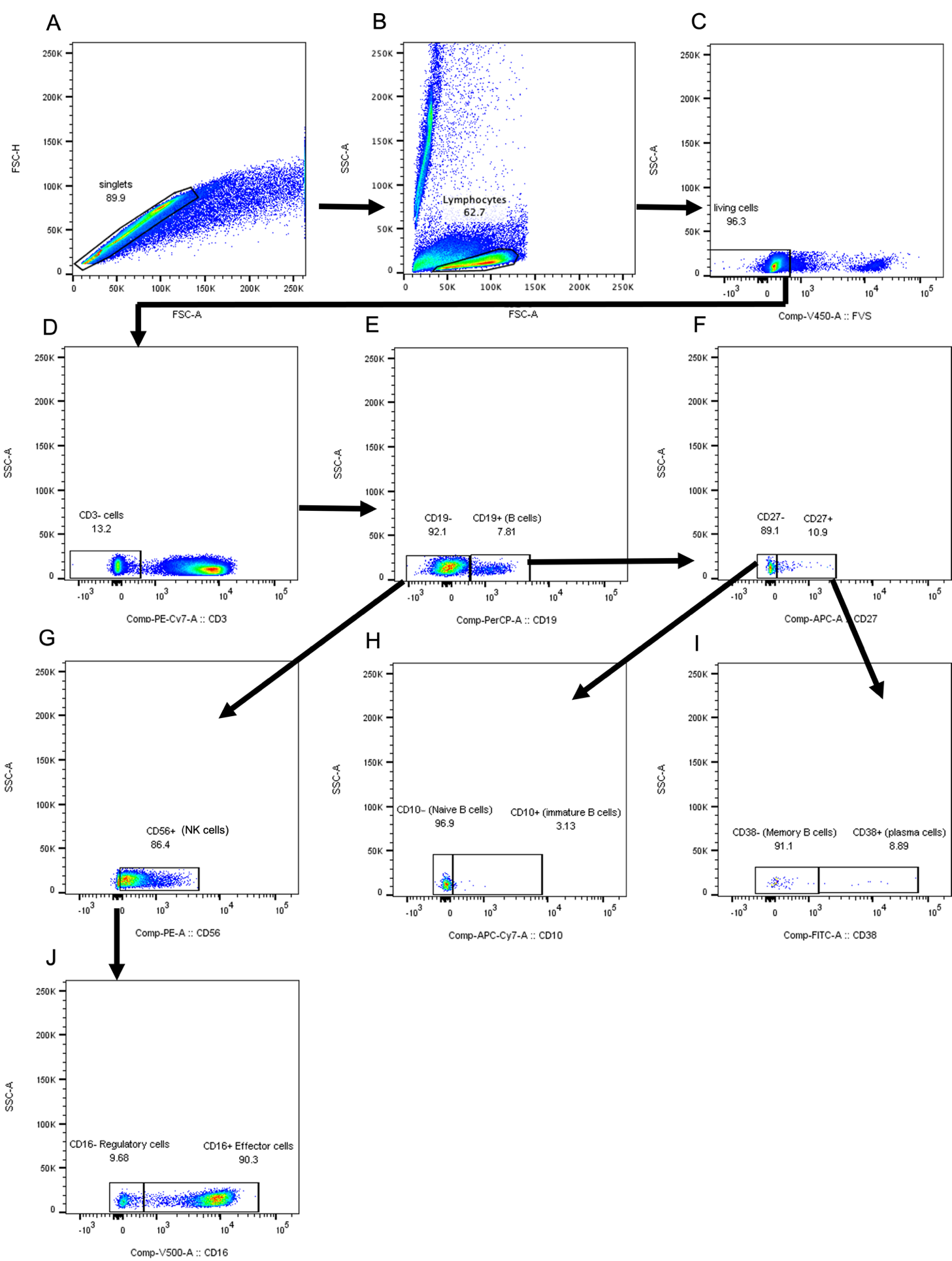
*

*Representative flow cytometry analysis with a sample from a healthy participant. Singlets were gated to discard doublets* ***(A)*** *and lymphocytes were identified using FSC versus SSC* ***(B).*** *Viable cells were selected* ***(C).*** *All CD3- cells were gated* ***(D)*** *and assessed for CD19 expression* ***(E).*** *CD19+ cells were defined as B cells. B cells were examined for CD27 expression* ***(F).*** *CD27- cells were examined for CD10 expression to define CD10- Naïve B cells and CD10+ Immature B cells (H). CD27+ cells were examined for CD38 expression, to define CD38- Memory B cells and CD38+ Plasmablasts* ***(I).*** *CD19- cells were then examined for CD56 expression* ***(G).*** *CD56+ cells were defined as NK cells, and examined for CD16 expression* ***(J),*** *to identify CD16- Regulatory and CD16+ Effector NK cells.*
